# Developing prediction models to estimate the risk of two survival outcomes both occurring: A comparison of techniques

**DOI:** 10.1002/sim.9771

**Published:** 2023-05-23

**Authors:** Alexander Pate, Matthew Sperrin, Richard D. Riley, Jamie C. Sergeant, Tjeerd Van Staa, Niels Peek, Mamas A. Mamas, Gregory Y. H. Lip, Martin O'Flaherty, Iain Buchan, Glen P. Martin

**Affiliations:** ^1^ Division of Informatics, Imaging and Data Science, Faculty of Biology, Medicine and Health University of Manchester, Manchester Academic Health Science Centre Manchester UK; ^2^ Institute of Applied Health Research University of Birmingham Birmingham UK; ^3^ Centre for Epidemiology Versus Arthritis, Centre for Musculoskeletal Research, Manchester Academic Health Science Centre University of Manchester Manchester UK; ^4^ Centre for Biostatistics, Manchester Academic Health Science Centre University of Manchester Manchester UK; ^5^ Keele Cardiovascular Research Group Keele University Stoke‐on‐Trent UK; ^6^ Liverpool Centre for Cardiovascular Science at University of Liverpool Liverpool John Moores University and Liverpool Heart & Chest Hospital Liverpool UK; ^7^ Department of Clinical Medicine Aalborg University Aalborg Denmark; ^8^ Institute of Population Health, Faculty of Health and Life Sciences University of Liverpool Liverpool UK

**Keywords:** clinical prediction model, multiple outcome, multivariate, simulation, survival analysis, time‐to‐event

## Abstract

**Introduction:**

This study considers the prediction of the time until two survival outcomes have both occurred. We compared a variety of analytical methods motivated by a typical clinical problem of multimorbidity prognosis.

**Methods:**

We considered five methods: product (multiply marginal risks), dual‐outcome (directly model the time until both events occur), multistate models (msm), and a range of copula and frailty models. We assessed calibration and discrimination under a variety of simulated data scenarios, varying outcome prevalence, and the amount of residual correlation. The simulation focused on model misspecification and statistical power. Using data from the Clinical Practice Research Datalink, we compared model performance when predicting the risk of cardiovascular disease and type 2 diabetes both occurring.

**Results:**

Discrimination was similar for all methods. The product method was poorly calibrated in the presence of residual correlation. The msm and dual‐outcome models were the most robust to model misspecification but suffered a drop in performance at small sample sizes due to overfitting, which the copula and frailty model were less susceptible to. The copula and frailty model's performance were highly dependent on the underlying data structure. In the clinical example, the product method was poorly calibrated when adjusting for 8 major cardiovascular risk factors.

**Discussion:**

We recommend the dual‐outcome method for predicting the risk of two survival outcomes both occurring. It was the most robust to model misspecification, although was also the most prone to overfitting. The clinical example motivates the use of the methods considered in this study.

## 1 BACKGROUND

Prognostic clinical prediction models (CPMs) use information that is available about an individual to estimate the risk of a future clinical event.^1^, ^2^, ^3^ Often, the outcome being predicted is a time‐to‐event, such as time‐to‐diagnosis of cardiovascular disease. Most CPMs developed in the literature focus on predicting only a single outcome.^4^, ^5^, ^6^, ^7^, ^8^, ^9^, ^10^, ^11^, ^12^ However, this is a suboptimal approach when clinical action relies on the likely prognosis across multiple events/outcomes. For example, consider CHADS_2_ and the CHA_2_DS_2_‐VASc scores^13^, ^14^ which estimates the risk of stroke in patients with atrial fibrillation and can be used to help decide whether to prescribe oral anti‐coagulants. The risk of stroke is balanced against the risk of major bleeding when on anti‐coagulants, which can be calculated using the HAS‐BLED score.^15^ These risks are also non‐static and alter with ageing and incident comorbidities.^16^, ^17^ Here, one is interested in predicting the risk of the outcomes both occurring (also referred to as the risk of “both‐of‐two” outcomes). A second example where modeling the risk of two survival outcomes both occurring is required is the prediction of local recurrence and distant metastasis of cancer, where clinical actions can depend on these developing in isolation or together (and in which order).^18^, ^19^, ^20^, ^21^ A third example is prediction of multimorbidity risk, which is becoming more prevalent in many countries with people living longer with more than one long‐term condition,^22^, ^23^, ^24^, ^25^, ^26^, ^27^ which is having a major impact on health systems globally.^28^, ^29^, ^30^, ^31^, ^32^, ^33^, ^34^ While there is a plethora of CPMs developed to predict risks of common noncommunicable diseases, these are each developed in isolation, meaning they cannot appropriately model the risk of multiple long‐term conditions occurring together. Also, many comorbidities tend to cluster together, as well as alter over time with ageing and changes in risk factors, with implications for clinical outcomes such as stroke and bleeding.^35^, ^36^ Thus, such risk estimation would allow policy makers to predict future levels of multimorbidity in the population and target resources accordingly, for example, to preventive measures for key comorbidities.

A potential reason for the lack of such risk prediction in practice is that it is currently unclear which of the available methods has the best predictive performance. The simplest approach is to develop univariate survival CPMs for each outcome separately. If there is no residual correlation between the outcomes at all‐time points (after conditioning on predictor variables), it is appropriate to multiply the corresponding risk scores from the univariate models to obtain the desired risk. However, such risk estimates will be miscalibrated if the (conditional) independence assumption does not hold. This concept is formally motivated in the following section. The extent of this miscalibration and how it may impact clinical practice is not clear. Currently, no study has been undertaken to compare the methods that can model the risk of both outcomes occurring when this assumption is violated.

Therefore, the aim of this study was 2‐fold: (1) to measure the extent of the miscalibration in prediction of the risk of both‐of‐two survival outcomes using univariate models, when there is residual correlation in the outcomes and (2) to compare the performance of a variety of methods that could be used for predicting the risk of both‐of‐two survival outcomes. Section 2 outlines each of the prediction approaches considered in this study. Section 3 contains a simulation comparing the performance of these methods. Section 4 is a clinical example considering the prediction of cardiovascular disease (CVD) and type 2 diabetes (T2D). Section 5 contains a discussion of the results from the simulation and clinical example, and an overall discussion and recommendations.

## 2 METHODS TO PREDICT THE RISK OF TWO SURVIVAL OUTCOMES BOTH OCCURRING

This section contains a summary of each method and how they can be used to estimate the risk of two survival outcomes both occurring. Note that the focus of this study is on prediction of the risk of outcomes that do not prevent the other from happening (ie, non‐competing events). The prediction of time‐to‐event outcomes in the presence of a competing risk has been covered extensively^37^, ^38^, ^39^, ^40^, ^41^, ^42^, ^43^, ^44^ and will not be the focus of this article.

### 2.1 Notation and preliminaries

Let $T_{A}$ and $T_{B}$ be the event times for two (non‐competing) events A and B, and $T_{C}$ be the time until censoring. For each individual we observe $T_{A^{*}}=\min\left\{T_{A},T_{C}\right\}$ and $T_{B^{*}}=\min\left\{T_{B},T_{C}\right\}$. Let $\delta_{A}$ be the event indicator for outcome A, such that $\delta_{A}=1$ if $T_{A}=T_{A^{*}}$, otherwise $\delta_{A}=0$. $\delta_{B}$ is defined similarly. Let X be a vector of baseline predictor variables, each of which might be predictive of A or B (or both). We assume one is primarily interested in estimating the risk of outcomes A and B occurring before timepoint t, given X; that is, $P\left(T_{A}\leq t,T_{B}\leq t|X\right)$. However, each of the methods can provide varying levels of insight beyond estimating this quantity, such as estimating marginal risk scores, the level of dependence between the two outcomes or the temporal ordering of events. We discuss the extra utility of each method in the discussion (Section 5.3). We assume a common censoring mechanism to both outcomes throughout this study which is likely to hold in the majority of scenarios, however all the following models can still be applied when A and B have different censoring mechanisms. We assume the censoring process is independent of A and B given X, and discuss the ability of the methods to account for informative censoring and implement competing risks analysis (with respect to a third competing event) in Section 2.7.

### 2.2 The product method

The product method is the most straightforward approach. One first develops univariate models for each outcome individually. We used Cox models in this study, but any time‐to‐event model could be used (eg, flexible parametric survival models). Univariate models for A and B allow the estimation of the marginal survival functions, $P\left(T_{A}>t|X\right)$ and $P\left(T_{B}>t|X\right)$, and the marginal risks $P\left(T_{A}\leq t|X\right)=1-P\left(T_{A}>t|X\right)$ and $P\left(T_{B}\leq t|X\right)=1-P\left(T_{B}>t|X\right)$. Under the assumption of conditional independence of A and B given X, the product of these will be an unbiased estimator of the risk.

$$P\left(T_{A}\leq t,T_{B}\leq t|X\right)=P\left(T_{A}\leq t|X\right)*P\left(T_{B}\leq t|X\right).$$

However, as the level of residual correlation increases, miscalibration of the product method in estimating $P\left(T_{A}\leq t,T_{B}\leq t|X\right)$ will increase; we examine the extent of this in the simulation study.

### 2.3 Dual‐outcome approach

The second method is to re‐define the outcome as being the time until both outcome events have occurred, and develop a univariate model to predict this new “dual‐outcome”. Let $T_{\mathrm{AB}}=\max\left\{T_{A},T_{B}\right\}$, and $T_{{\mathrm{AB}}^{*}}=\min\left\{T_{\mathrm{AB}},T_{C}\right\}$, and $\delta_{\mathrm{AB}}=1$ if $T_{\mathrm{AB}}=T_{{\mathrm{AB}}^{*}}$, otherwise $\delta_{\mathrm{AB}}=0$. Then a univariate model (Cox proportional hazards model or otherwise) can be developed on AB to estimate:

$$P\left(T_{\mathrm{AB}}\leq t|X\right)=1-P\left(T_{\mathrm{AB}}>t|X\right).$$

Given that $\left\{T_{\mathrm{AB}}\leq t\right\}$ ⇔ $\left\{T_{A}\leq t,T_{B}\leq t\right\}$, then:

$$P\left(T_{A}\leq t,T_{B}\leq t|X\right)=P\left(T_{\mathrm{AB}}\leq t|X\right).$$

Therefore, the dual‐outcome approach can provide estimates of $P\left(T_{A}\leq t,T_{B}\leq t|X\right)$ but does not have the ability to calculate marginal risk scores for each outcome in isolation.

### 2.4 Copulas

Copulas are implemented by defining a dependence structure between two marginal cumulative distribution functions. The general framework is not restricted to survival models, but they have garnered a lot of attention in this area. For two survival outcomes, A and B, the survival function for both outcomes is defined as:

$$P\left(T_{A}>t,T_{B}>t|X\right)=C_{\theta }\left\{P\left(T_{A}>t|X\right),P\left(T_{B}>t|X\right)\right\},$$

where $C_{\theta }$ is the bivariate copula, a function of a parameter θ that represents the degree of dependence between A and B. After a given copula has been chosen, the parameter θ is estimated by either estimating the parameters from the marginal distributions, and then estimating the copula parameter(s) (the two‐step approach),^45^ or a joint likelihood can be maximized to estimate the marginal likelihood parameters and the copula parameter simultaneously.^46^, ^47^, ^48^

Some common examples of bivariate copulas (as given by Emura et al^49^) are:

The independence copula:

C(u,v)=uv.

The Clayton copula:

$$C_{\theta }\left(u,v\right)={\left(u^{-\theta }+v^{-\theta }-1\right)}^{-1/\theta },\theta >0.$$

The Gumbel copula:

$$C_{\theta }\left(u,v\right)=\exp\left[-{\left\{{\left(-\log\left(u\right)\right)}^{\theta +1}+{\left(-\log\left(v\right)\right)}^{\theta +1}\right\}}^{\frac{1}{\theta +1}}\right],\theta \geq 0.$$

The Farlie‐Gumbel‐Morgenstern (FGM) copula:

$$C_{\theta }\left(u,v\right)=\mathrm{uv}\left\{1+\theta \left(1-u\right)\left(1-v\right)\right\},-1\leq \theta \leq 1.$$

An explanation of copulas for multivariate survival analysis is given by Georges et al,^50^ as well as concise summaries by Govindarajulu and D'Agostino,^43^ and comprehensively covered in the books by Nelsen^51^ and Emura et al.^49^ Note that these references are concerned with modeling the survival function $P\left(T_{A}>t,T_{B}>t|X\right)=C_{\theta }\left\{P\left(T_{A}>t|X\right),P\left(T_{B}>t|X\right)\right\}$, whereas we are interested in estimating the risk $P\left(T_{A}\leq t,T_{B}\leq t|X\right)$. This risk can be estimated using the following equation:

$$P\left(T_{A}\leq t,T_{B}\leq t|X\right)=1-P\left(T_{A}>t|X\right)-P\left(T_{B}>t|X\right)+C_{\theta }\left\{P\left(T_{A}>t|X\right),P\left(T_{B}>t|X\right)\right\}.$$

A simple proof of this equation is given in supporting information file 1.

An advantage of using copulas for risk prediction is that they explicitly model the association between the outcomes, providing a very clear framework in which to model the dependence between the outcomes. A potential drawback is that a parametric correlation structure (the copula itself) must be assumed, meaning the results may be sensitive to the choice of copula. This is something we explore through the simulation in Section 3. Note that implementing the independence copula would be analogous to the product method. To fit the copula models we implemented the joint estimation approach of Marra et al,^48^ implemented in the package GJRM.^52^, ^53^

### 2.5 Frailty models

A frailty model is a survival model with a random effect term to account for unexplained heterogeneity in survival times.^54^, ^55^, ^56^ Shared frailty models are generally applied to data which has a multilevel structure.^57^ We propose that shared frailty models could be used to model the dependence between two outcomes, and subsequently the risk of them both occurring. To do this using a Cox framework, the following model would be fit (introducing subscript i to denote individual i):

$$h_{A}\left(t|\omega_{i}\right)=\omega_{i}h_{0,A}\left(t\right)\exp\left(\beta_{A}X_{i}\right),$$

$$h_{B}\left(t|\omega_{i}\right)=\omega_{i}h_{0,B}\left(t\right)\exp\left(\beta_{B}X_{i}\right),$$

where $\omega_{i}$ is the shared random effect for individual i, which could have a gamma or lognormal distribution. Here, the distribution of $\omega_{i}$ models the association between the distinct survival processes, thereby handling the correlation and enabling risk prediction of both outcomes.

To fit this model in practice, the datasets for each outcome must be stacked on top of each other, and the following shared frailty model fit to the data:

$$h\left(t|\omega_{i}\right)=\omega_{i}h_{0}\left(t\right)\exp\left(\beta_{\mathrm{ind}}*I\left[X_{\mathrm{ind}}=A\right]+\beta_{A}X_{i}*I\left[X_{\mathrm{ind}}=A\right]+\beta_{B}X_{i}\right),$$

where $X_{\mathrm{ind}}\in \left\{A,B\right\}$ is an indicator variable denoting which outcome the row corresponds to, $\beta_{B}$ is the hazard ratios shared across both outcomes, and $\beta_{A}$ tests whether the hazard ratios for outcome A differ from the hazard ratios of outcome B ($\beta_{A}=0$ implies no change in hazard ratios). Note that this approach relies on the baseline hazards of the two survival processes being proportional, $h_{0,B}\left(t\right)=h_{0}\left(t\right),h_{0,A}\left(t\right)=h_{0}\left(t\right)*\exp\left(\beta_{\mathrm{ind}}\right)$. To alleviate this assumption, a stratified model could be fit:

$$h\left(t|\omega_{i}\right)=\omega_{i}h_{j}\left(t\right)\exp\left(\beta_{A}X_{i}*I\left[X_{\mathrm{ind}}=A\right]+\beta_{B}X_{i}\right)$$

for j∈{A,B}. This model allows the estimation of marginal risk scores given the random effect $P\left(T_{A}\leq t|X_{i},\omega_{i}\right)=1-P\left(T_{A}>t|X_{i},\omega_{i}\right)$ and $P\left(T_{B}\leq t|X_{i},\omega_{i}\right)=1-P\left(T_{B}>t|X_{i},\omega_{i}\right)$. The observations within a cluster in a shared frailty model are assumed to be independent after conditioning on the random effect, meaning the risk can be estimated as:

$$P\left(T_{A}\leq t,T_{B}\leq t|X_{i},\omega_{i}\right)=P\left(T_{A}\leq t|X_{i},\omega_{i}\right)*P\left(T_{B}\leq t|X_{i},\omega_{i}\right).$$

To estimate the risk for new individuals, one needs to integrate over the distribution of ω:

$$P\left(T_{A}\leq t,T_{B}\leq t|X_{i}\right)=\int P\left(T_{A}\leq t|X_{i},\omega \right)*P\left(T_{B}\leq t|X_{i},\omega \right)f_{\omega }\left(\omega \right)d\omega ,$$

where $f_{\omega }\left(\omega \right)$ is the estimated probability density function for ω.

There are some similarities, and also key differences, between frailty models and copula models which are discussed elsewhere.^46^

### 2.6 Multistate models

**FIGURE 1:**
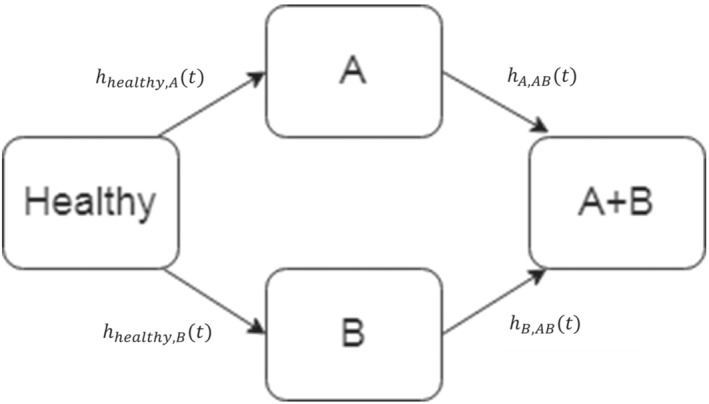
A multistate model for predicting two outcomes *A* and *B*

In multistate models each outcome event is seen as a state, and the probability of transitioning between different states is modeled using competing risks approaches.^40^ Generally, cause‐specific hazard functions are calculated for each transition, which can be done parametrically or semi‐parametrically (ie, Cox proportional hazards). A diagram representation of a model for predicting two outcomes is given in Figure 1, where h(t) represents the hazard rate for each transition. It is important to note that here we are working in a context of competing risks, but the goal is not to estimate competing risks scores (although this is possible within this framework, see Section 2.7). The target estimand is still $P\left(T_{A}\leq t,T_{B}\leq t|X\right)$.

After the cause‐specific hazards have been fitted, risk estimates can be calculated using the transition probabilities, which is the probability of being in a given state at time t, when in a given state at time s. The risk of both outcomes can be calculated as the probability of being in the “*A* + *B*” at time t, when in the healthy state at time 0. There are several ways to estimate the transition probabilities depending on whether the Markov assumption holds or not.^58^, ^59^, ^60^, ^61^, ^62^ However, if all individuals start in the initial state, and one is only interested in transition probabilities out the initial state at time 0 (which is the case for this study), then the transition probabilities are equivalent to the “state occupational probabilities,” and the Aalen‐Johansen (the simplest approach) will be a consistent estimator even for non‐Markov data. A detailed description of how to fit multistate models using the package mstate is given by de Wreede et al.^63^, ^64^

### 2.7 Development of competing risks models

A key aspect to discuss, in the context of multiple time‐to‐event outcomes, is competing risks.^41^, ^42^, ^43^, ^44^ Throughout this article, we assume that the two events (*A* and *B*) do not prevent each other, motivated by examples as given in the introduction. However, there may be a third event *D* (e.g. death) which does act as a competing risk for both *A* and *B*. While competing risks approaches are well developed for univariate analyses, this is not the case for some of the other methods described above.

For the product method, univariate competing risks models can be fitted in R using the mstate package.^40^, ^63^, ^64^ These risks can then be multiplied to estimate the risk of both outcomes, in the same way that was done for the non‐competing risk scores. Multistate models are implemented within a competing risks framework and therefore it is straightforward to produce estimates which account for a competing event by introducing an absorbing state which individuals may move into after the occurrence of the competing event. Theory on frailty models with competing risks is well developed,^65^, ^66^, ^67^, ^68^, ^69^ and more recently copula models with competing risks,^70^, ^71^, ^72^, ^73^, ^74^ but there is a lack of flexible publicly available software to implement these methods. The majority of the theory focuses on frailty terms shared between the competing risks, whereas in this study we are interested in a frailty term to estimate the risk of two outcomes both occurring in the presence of a third competing risk. We see no reason these methods would not extend to the setting in this study which is relatively simple in comparison. A straightforward method to account for a competing risk is to not censor individuals after the competing event occurs, instead setting the event time to the largest possible follow up time. They will then remain in the “at risk” group of individuals after the competing event, giving estimates of risk that account for the competing risk. The drawback of this approach is that the risk of the competing event itself cannot be calculated, which may or may not be of importance.

## 3 SIMULATION

We detail the methods for the simulation using the “aims, data‐generating mechanisms, estimands, methods, performance measures” (ADEMP) structure.^75^ Code for running the simulation is available from our GitHub public repository.^76^

### 3.1 Simulation aims and overview

The first aim of the simulation was to measure the extent of the miscalibration in the risk prediction of both‐of‐two survival outcomes using the product method, when there was residual correlation in the outcomes. The second aim was to compare the performance (calibration and discrimination) of available methods for predicting the risk of both‐of‐two survival outcomes.

When interest is in predicting the risk of both‐of‐two outcomes, the dual‐outcome approach seems the most natural approach, and motivation is required as to why the copula, frailty or msm approaches may outperform the dual‐outcome. All these methods make different assumptions about the underlying data. For example, the dual‐outcome relies on the survival outcome $T_{\mathrm{AB}}$ meeting the distributional assumptions of the chosen model. If cox regression is chosen, the hazard function for AB must meet the proportional hazards assumption, or for an accelerated failure time model, that the covariates act multiplicatively on the mean survival time. Given the complex form of the survival distribution and hazard function for the dual outcome, it is unlikely either of these assumptions would hold in practice (supporting information file 1). In contrast, the multistate model only makes these assumptions on the cause‐specific hazards for each outcome in isolation. Similarly, the frailty models and copula models specify the marginal distributions separately, and then place a parametric distribution on the residual correlation. These model specifications may therefore be more appropriate than those of the dual outcome. We therefore constructed our simulation scenarios around robustness to model misspecification, to assess performance in scenarios where data was generated under a different mechanism from the model being applied. To enable a comprehensive comparison, we generated data under a variety of data‐generating mechanisms, each matching the model structure of one of the analysis methods (full factorial design).

A second reason that the dual‐outcome method may be outperformed by the other methods is due to statistical power. The dual‐outcome approach discards some outcome event data, by ignoring events that occur on their own (ie, those patients that experience only one of the outcomes are not counted as “events”). The copula and frailty approaches estimate predictor coefficients for each marginal distribution separately and will therefore benefit from an increase in power when estimating these predictor coefficients. This may lead to lower levels of overfitting. The multistate model may suffer from a similar issue with respect to power, as only a small number of events will occur for transitions A→AB and B→AB. This may result in overfitting in the estimation of these cause‐specific hazards. This is a particular risk at small sample sizes or for rarer outcomes. To assess this, we varied both the sample size and marginal risk of each outcome in the simulation.

### 3.2 Data generation mechanisms

For each data generation mechanism (DGM), we simulated 1000 development datasets of size n, where n was 1000, 2500, or 5000, depending on simulation scenario. Baseline predictors were two random variables, $X_{1}˜N(0,1)$ and $X_{2}˜N(0,1)$.

#### 3.2.1 DGM‐1: Multistate model

We simulated data from the multistate model depicted in Figure 1, with exponential hazards:

$$h_{\text{healthy},A}\left(t\right)=\left(\frac{1}{\lambda_{A}}\right)*\exp\left(X.\beta_{A}\right),$$

$$h_{\text{healthy},B}\left(t\right)=\left(\frac{1}{\lambda_{B}}\right)*\exp\left(X.\beta_{B}\right),$$

$$h_{A,\mathrm{AB}}\left(t\right)=\left(\frac{1}{\lambda_{B}*\lambda_{A,B}}\right)*\exp\left(X.\beta_{B}\right),$$

$$h_{B,\mathrm{AB}}\left(t\right)=\left(\frac{1}{\lambda_{A}*\lambda_{B,A}}\right)*\exp\left(X.\beta_{A}\right).$$

We assume the shape of transition $h_{\text{healthy},A}\left(t\right)$ and $h_{B,\mathrm{AB}}\left(t\right)$ are the same as they are both transitions resulting in the development of outcome *A*. The term $\lambda_{B,A}$ causes a change in the scale of developing condition *A* (ie, the event to happen at a quicker or slower rate), once condition *B* has been developed. Similarly, $\lambda_{A,B}$ causes a change in the scale of developing condition *B* once condition *A* has been developed. These terms therefore controlled the level of residual correlation ($\lambda_{B,A}=\lambda_{A,B}=1$ being no residual correlation) and are therefore key parameters to vary in this simulation. $\beta_{i}$ is a vector containing the log‐hazard ratios of the effect of $X_{1}$ and $X_{2}$ on outcome i.

#### 3.2.2 DGM‐2 (Clayton), DGM‐3 (Gumbel) and DGM‐4 (Frank): Copulas

Let $C_{\theta }\left(u,v\right)$ be an copula. We first generated n sets of u∼Unif(0,1), v∼Unif(0,1) from this copula. We then generated event times from each marginal distribution separately, assuming that the random draws of u and v were from the cumulative distribution function for outcome A and B, respectively. We assumed survival times for A and B follow an exponential distribution with scales $\lambda_{A}$ and $\lambda_{B}$, respectively. Specifically, the hazard for *A* was:

$$h_{A}\left(t\right)=\left(\frac{1}{\lambda_{A}}\right)*\exp\left(X.\beta_{A}\right)$$

and the survival function was therefore:

$$S_{A}\left(t\right)=\exp\left(-H_{A}\left(t\right)\right)$$

$$=\exp\left(-\left(\frac{t}{\lambda_{A}}\right)*\exp\left(X.\beta_{A}\right)\right).$$

Then for a random event time $t_{A}$, we define $u=P\left(T_{A}>t_{A}\right)$, and calculate the simulated event time $t_{A}$ as follows:

$$u=S_{A}\left(t_{A}\right),$$

$$u=\exp\left(-\left(\frac{t_{A}}{\lambda_{A}}\right)*\exp\left(X.\beta_{A}\right)\right),$$

$$\lambda_{A}*\left(\frac{-\log\left(u\right)}{\exp\left(X.\beta_{A}\right)}\right)=t_{A}.$$

Therefore we simulated event times $t_{A}$ according to the distribution:

$$t_{A}˜\lambda_{A}*\left(\frac{-\log(u)}{\exp\left(X.\beta_{A}\right)}\right).$$

Event times $t_{B}$ were simulated similarly.

$$t_{B}˜\lambda_{B}*\left(\frac{-\log(v)}{\exp\left(X.\beta_{B}\right)}\right).$$

These event times will have marginal exponential distributions with $\lambda_{A}$ and $\lambda_{B}$, but will have a joint distribution that has the properties of the copula $C_{\theta }$.

The term θ induces residual correlation between the outcomes, and therefore the value of θ is of key interest in simulations under this DGM.

#### 3.2.3 DGM‐5 (log‐normal) and DGM‐6 (gamma): Frailty models

We first define two exponential baseline hazards for outcomes A and B, $h_{0,A}\left(t\right)$ and $h_{0,B}\left(t\right)$. For DGM‐5 generate a shared Gaussian frailty term for each individual, $\omega_{i}˜\text{lognormal}\left(1,\sigma^{2}\right)$. For DGM‐6 generate a shared gamma frailty term for each individual, $\omega_{i}˜\text{Gamma}\left(\frac{1}{\;\beta },\beta \right)$.

We then generate survival times according to the hazard functions $h_{A}\left(t|\omega_{i}\right)$ and $h_{B}\left(t|\omega_{i}\right):$

$$h_{A}\left(t|X,\omega_{i}\right)=\omega_{i}h_{0,A}\left(t\right)\exp\left(\beta_{A}X_{i}\right),$$

$$h_{B}\left(t|X,\omega_{i}\right)=\omega_{i}h_{0,B}\left(t\right)\exp\left(\beta_{B}X_{i}\right).$$

The term $\omega_{i}$ induces residual variation between the outcomes, and therefore the variance of this parameter is of key interest in simulations under this DGM. A variance of zero (θ=0 or β→0) will result in no residual correlation, and as the variance increases this will result in more residual correlation.

#### 3.2.4 Simulation scenarios and choice of input parameters

The choice of simulation scenarios was based around the aims of the simulation outlined in Section 3.1. The three major aspects of the simulation that were varied were (i) DGM; to assess each methods sensitivity to model misspecification, (ii) the level of residual correlation; this quantity drives the bias in the product method and is the reason we must use these alternative approaches, and (iii) the amount of statistical power available to predict the outcome; this may also drive model performance.

We have outlined the 6 DGMs in Sections 3.2.1 to 3.2.4. We created a further six scenarios: lower marginal risks, no residual correlation (LN); lower marginal risks, lower residual correlation (LL); lower marginal risks, higher residual correlation (LH); higher marginal risks, no residual correlation (HN); higher marginal risks, lower residual correlation (HL); and higher marginal risks, higher residual correlation (HH), based off the amount of residual correlation and incidence of the outcomes.

The lower marginal risk scenarios (LN, LL, and LH) were targeted to have marginal risks of outcome A (10%) and B (10%) in line with the marginal risks of the outcomes in the clinical example from Section 4. These were increased to a marginal risk of 30% for each outcome in the higher marginal risk scenarios (HN, HL, and HH). The lower residual correlation scenarios (LL and HL) were targeted to have a true mean risk 20% higher than the mean risk when assuming independence after conditioning on predictor variables. This was increased to 50% for the higher residual correlation scenarios (LH and HH). The targeted values for each scenario are displayed in Table 1. We aimed to keep the magnitude of predictor coefficients similar across each DGM within a given scenario. The exact input parameters for each scenario, and a more detailed explanation of the process for choosing these parameters, are provided in supporting information file 1.

**TABLE 1:** Targeted values of marginal risks and risks of both outcomes occurring for each simulation scenario

	Scenario LN	Scenario LL	Scenario LH	Scenario HN	Scenario HL	Scenario HH
Marginal risk *A*	10%	10%	10%	30%	30%	30%
Marginal risk *B*	10%	10%	10%	30%	30%	30%
${\mathrm{jr}}_{\mathrm{ind}}$	1%	1%	1%	9%	9%	9%
${\mathrm{jr}}_{\text{pred}}$	1.25%	1.25%	1.25%	11.25%	11.25%	11.25%
${\mathrm{jr}}_{\text{true}}$	1.25%	1.5%	1.875%	11.25%	13.5%	16.875%
${\mathrm{jr}}_{\text{true}}/{\mathrm{jr}}_{\text{pred}}$	1 (0% increase)	1.2 (20% increase)	1.5 (50% increase)	1 (0% increase)	1.2 (20% increase)	1.5 (20% increase)

*Note*: $\;{\mathrm{jr}}_{\mathrm{ind}}$ = mean risk in population assuming complete independence (no conditioning on predictors); ${\mathrm{jr}}_{\text{pred}}$ = mean risk in population assuming independence after conditioning on available predictors; ${\mathrm{jr}}_{\text{true}}$ = true mean risk in population; process for calculating ${\mathrm{jr}}_{\mathrm{ind}}$, ${\mathrm{jr}}_{\text{pred}}$, and ${\mathrm{jr}}_{\text{true}}$ are given in supporting information file 1.

Finally, we created more scenarios based on development dataset sizes of n∈{1000,2500,5000}. The minimum sample size (1000) was estimated using the sample size formula of Riley et al,^77^ for a risk prediction model predicting the dual‐outcome in scenario LN. A conservative estimate of $R_{\mathrm{CS}\text{\_}\mathrm{adj}}^{2}$ equivalent to an $R_{\text{Nagelkerke}}^{2}$ of 0.15 was used in the calculation, giving a minimum required sample size of 896. We therefore chose N=1000 as the smallest sample size in the simulation. Code for this step is provided on GitHub.^76^ This resulted in a total of 6*4*3 + 2*3 = 78 scenarios (note that for no residual correlation scenarios LN and HN we do not generate data using every DGM). The input parameters we have chosen to vary are based on the aims of the simulation. A censoring time was simulated from a survival distribution with an exponential hazard, and log hazard ratios of 0.1 for both $X_{1}$ and $X_{2}$. The rate was chosen to target 5% of the evets to be censored in the lower marginal risk scenarios (see supporting information file 1 for exact values).

When interpreting the results, it is important to note that for a given DGM, each misspecified model may be a different “distance” away from the model used to generate the data. If two models are very dissimilar, we would expect poor performance for either of these models when the other is used for the DGM. If a given model has very poor performance across a range of the DGMs, it is likely quite “far” from all the other model structures, and will be deemed sensitive to model misspecification.

### 3.3 Estimands and other targets

The main estimand was the set of points $P\left(T_{A}\leq 3653,T_{B}\leq 3653|X\right)$ over all individuals in the validation cohort. This is the risk of developing outcome A and B, prior to time t=3653, which corresponds to 10‐year risk (assuming an integer to be 1 day) in line with the clinical example. A further target of the simulation was to report on the discrimination of each model in the validation cohort.

### 3.4 Methods

Each of the methods outlined in Section 2 was used to estimate $P\left(T_{A}\leq t,T_{B}\leq t|X\right)$, as described in Section 2. The estimated risks of developing both A and B are hereto referred to as ${\text{risk}}_{\text{product}}$ (product of univariate models), ${\text{risk}}_{d-o}$ (dual‐outcome model), ${\text{risk}}_{\mathrm{cop}-\text{clay}}$, ${\text{risk}}_{\mathrm{cop}-\mathrm{gum}}$, ${\text{risk}}_{\mathrm{cop}-\mathrm{FGM}}$ (copula model assuming Clayton, Gumbel, or FGM copulas), ${\text{risk}}_{\text{frail}-\mathrm{norm}}$, ${\text{risk}}_{\text{frail}-\mathrm{gam}}$ (frailty model assuming log‐normal or gamma frailty distribution), ${\text{risk}}_{\mathrm{msm}}$ (multistate model).

The dual‐outcome model was fitted using a Cox proportional hazards model given this is the most common approach in practice. It was therefore important to test its performance. Alternative parametric approaches could be used to remove reliance on the proportional hazards assumption, but will each have their own set of distributional assumptions. The frailty models were fitted using a Bayesian MCMC approach utilizing the rstan package.^78^ A Weibull baseline hazard was assumed, but any distribution could be used. Code for this is available from our GitHub public repository.^76^ This approach was used as the likelihood was very flat and convergence issues were encountered when attempting to fit these models using maximum likelihood or expectation maximization algorithms.

### 3.5 Performance measures

We compared each method's ability to estimate the set of points $P\left(T_{A}\leq 3653,T_{B}\leq 3653|X\right)$ by assessing moderate calibration.^79^ Calibration was assessed in a validation cohort of size 1000 generated using the same DGM as the development dataset. A new validation dataset was simulated for every development dataset. We generated flexible calibration curves by regressing the true risks on the predicted risks using a linear model with multiple fractional polynomials. True risks under each DGM were calculated using the process given in supporting information file 1. While in practice one would regress on the observed outcomes themselves, the simulation allows us to regress on the underlying true risk, thereby giving a more accurate assessment of calibration. The resulting curves gave the observed (true) risk as a function of predicted risk. We report the (pointwise) median and 5th/95th percentile of the calibration plots across the 1000 simulation iterations. Throughout this article, we refer to the median calibration curve and 5 to 95 percentile range in calibration curves across the 1000 iterations as “average calibration” and “calibration variation,” respectively. A detailed process for producing these calibration plots is given in supporting information file 1. Discrimination was assessed in the validation cohort using Harrell's C statistic.^80^

There are two points of clarification with regards to calibration as a performance measure. First, note that calibration is defined as the difference between the predicted risks and observed risks (or event rates) in a cohort of interest. In this simulation, the estimand itself are the “observed risks,” and the predicted risks from each method are the “predicted risks.” We therefore reason that the average calibration curve in this simulation is analogous to bias. Similarly, the calibration variation is analogous to the SE of the estimator of the risk. Second, note that the estimand in this simulation is a set of points. Therefore rather than presenting the bias in the estimation of a single estimand, our bias is presented as a line over the range of predicted risks. There is also a different level of variability at each point along this line. For example when individuals have very low predicted risks (near 0), we will expect to see less calibration variation than when predicted risks are bigger.

### 3.6 Simulation results

#### 3.6.1 Discrimination

For all scenarios the discrimination of all methods were similar (supporting information file 2: Tables S4.1‐S4.14), meaning each methods ability to risk‐rank individuals was similar. For scenarios LN and LL, *N* = 1000 only, there was a small drop in the discrimination of the dual‐outcome and msm methods. This simplifies the discussion greatly. Both calibration and discrimination are seen as highly valuable performance metrics to report.^1^, ^2^, ^81^, ^82^ However each may be more important in different clinical settings. For example, if the clinical strategy is to treat all individuals over a certain risk threshold with a low‐cost intervention (such as statins), then calibration may be more important than discrimination. However, if there is limited resources and an intervention can only be given to a fixed number of individuals who would all benefit from the treatment (say a diagnostic operation), then discrimination is arguably more important, to ensure the highest risk individuals receive the treatment first. Given the similar level of discrimination of every method, we do not have to weigh up the importance of calibration vs discrimination, as there are no scenarios where a method outperforms the others with respect to calibration but performs worse with respect to discrimination. We therefore focus on the calibration for the remainder of the results section.

#### 3.6.2 Calibration

**FIGURE 2:**
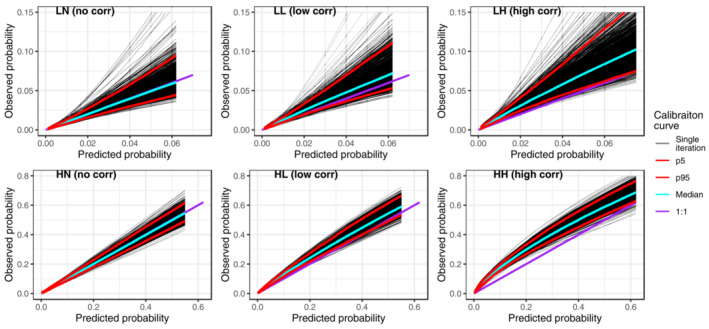
Calibration curves of the product method across the 1000 simulations, paneled by simulation scenario (*N* = 1000). Blue line = median calibration curve; red lines = fifth and 95th percentile in calibration curves. For scenarios LL, LH, HL and HH, the clayton DGM (DGM‐2) has been used

To answer the first aim of this study, we present the calibration curves of the product method for varying levels of residual correlation (Figure 2). When there was no residual correlation (scenarios LN and HN), the product method was well calibrated on average across the entire range of predicted risk. However as residual correlation increased (scenarios LL, LH, HL, and HH) the product method became increasingly miscalibrated, often underpredicting the risk. This was consistent regardless of the sample size of the development cohort (supporting information file 2: Figures S1.1‐S1.3).

To answer the second aim of this study, we now present the average calibration, and calibration variation of all the analysis methods over a range of scenarios and sample sizes. We have selected a subset of results to present in the main article, however equivalent plots for all scenarios and sample sizes are available in supporting information file 2. We present the results in line with key aspects of the simulation that were outlined in Section 3.2.4. For the rest of this section, when we refer to “all methods,” we are not considering the product method.

##### Impact of model misspecification

**FIGURE 3:**
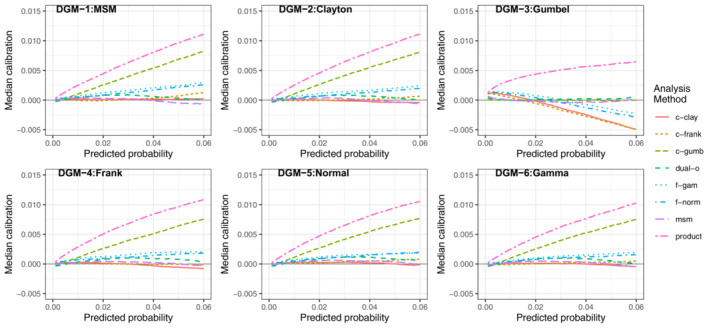
Median calibration (observed−predicted risk) curves across the 1000 simulations for scenario LL (lower outcome prevalence, lower residual correlation), *N* = 5000

The dual‐outcome and msm methods had the most consistent performance across all the DGMs, indicating they were the most robust to model misspecification. For example, in scenario LL when *N* = 5000 (Figure 3), the msm and dual‐outcome methods had extremely good average calibration across every DGM, demonstrating their ability to handle model misspecification. The Clayton and Frank copula models also had very good average calibration for 5 of the DGMs but were very poorly calibrated under the Gumbel DGM. On the contrary, the Gumbel copula method was very poorly calibrated under all DGMs except the Gumbel DGM. The normal and gamma frailty models had good average calibration across all DGMs, but not as strong as the msm or dual‐outcome methods.

**FIGURE 4:**
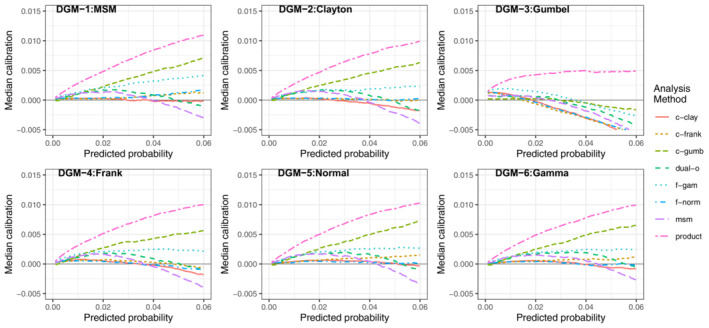
Median calibration (observed−predicted risk) curves across the 1000 simulations for scenario LL (lower outcome prevalence, lower residual correlation), *N* = 1000

For scenario LL when *N* = 1000 (Figure 4), the average calibration of the msm and dual‐outcome methods were poor across all DGMs (more so for the msm) and showed signs of overfitting; under prediction at lower predicted risks and over prediction at higher predicted risks. However, it should be noted that performance was consistent across all DGMs, supporting the idea that the both models are robust to model misspecification. The average calibration of the other methods (Clayton, Frank and Gumbel copula models, and normal and gamma frailty models) had a similar pattern to when *N* = 5000. All methods (except the Gumbel copula) had poor average calibration under the Gumbel DGM and had good average calibration across the other 5 DGMs, with performance particularly strong when each model was correctly specified. The Gumbel model had very poor average calibration under all DGMs except the Gumbel DGM. This indicates that the frailty and copula methods are more sensitive to model misspecification, in particular the Gumbel copula.

##### Impact of increasing sample size or marginal risks

As noted in the previous section, as the sample size decreased (Figures 3 and 4) the msm and dual‐outcome were more prone to overfitting than the frailty or copula models. The similarity in average calibration between *N* = 1000 and 5000 for the frailty and copula methods indicates that while they are more sensitive to model misspecification, they are less data intensive approaches.

**FIGURE 5:**
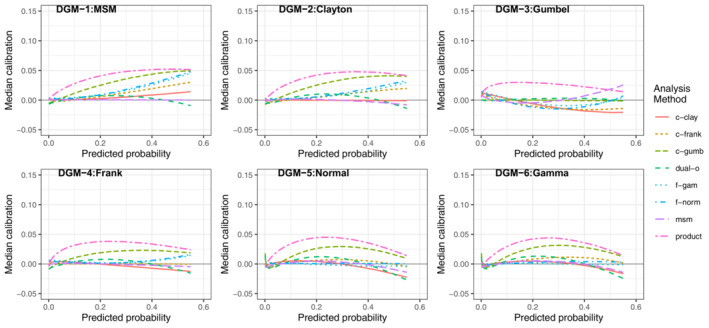
Median calibration (observed−predicted risk) curves across the 1000 simulations for scenario HL (higheroutcome prevalence, lower residual correlation), *N* = 5000

**FIGURE 6:**
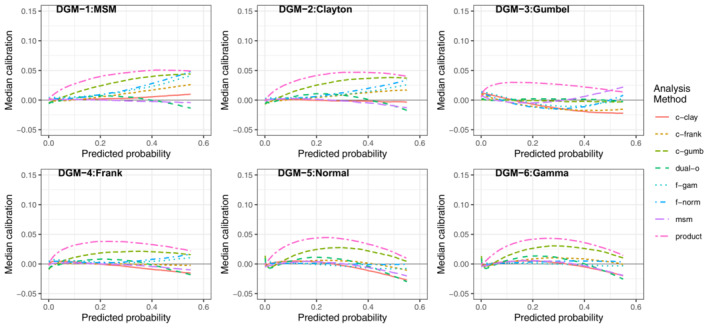
Median calibration (observed−predicted risk) curves across the 1000 simulations for scenario HL (higher outcome prevalence, lower residual correlation), *N* = 1000

When the incidence of both outcomes increased (but keeping the same level of residual correlation)—that is comparing scenario HL (Figures 5 and 6) with scenario LL (Figures 3 and 4)—all methods were generally better calibrated at lower sample sizes. This was highlighted by there being less of a difference in performance between *N* = 5000 (Figure 5) and *N* = 1000 (Figure 6); that is, the required sample size to mitigate overfitting decreased. When the marginal incidence was higher, the msm and dual‐outcome were less impacted by the smaller sample size (Figure 6), retaining good calibration for *N* = 1000.

**FIGURE 7:**
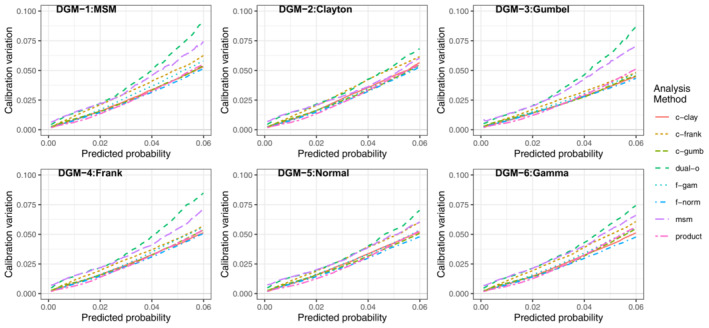
5 to 95 percentile range of calibration curves across the 1000 simulations for scenario LL (lower outcome prevalence, lower residual correlation), *N* = 1000)

Figure 7 plots the 5 to 95 percentile range in calibration curves of each method against the predicted risk in scenario LL at *N* = 1000. The value of the y‐axis is analogous to the distance between the blue lines (fifth percentile and 95th percentile) in Figure 2. As previously mentioned, the level of variation in the calibration curves increases when the predicted risks get bigger. The msm and dual‐outcome had the largest variability in calibration across every DGM, with little to choose between the other methods. The Frank copula method generally had the third highest calibration variation. This greater calibration variation for msm and dual‐outcome supports the idea that they are more data intensive approaches. These results held across all scenarios and sample sizes (supporting information file 2: Figures S3.1‐S3.12), although as the sample size increased the absolute difference in calibration variation between all analysis methods became negligible.

##### Impact of increasing the residual correlation

We saw similar patterns in results for the scenarios with higher levels of residual correlation (supporting information file 2: Figures S2.4‐S2.6 and S2.10‐S2.12). The dual‐outcome and msm methods again had the most consistent median calibration across all DGMs and performed well at the higher sample sizes. The Clayton and Frank copula methods also had good median calibration, consistent with that of dual‐outcome and msm for the majority of DGMs. There were very high levels of miscalibration in the product method, to be expected given the higher levels of residual correlation.

**FIGURE 8:**
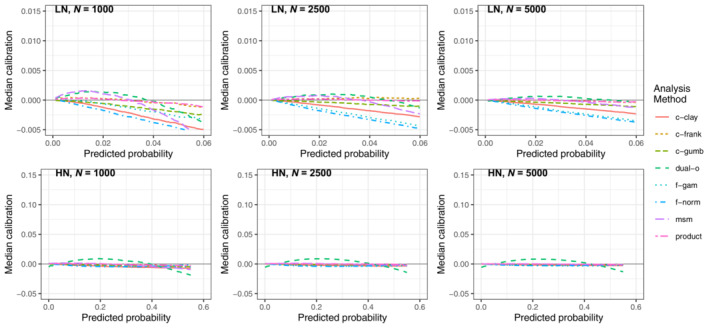
Median calibration curves (observed−predicted risk) across the 1000 simulations in the presence of no residual correlation (scenarios LN and HN), *N* = 1000, 2500, 5000. All the DGMs were equivalent in the presence of no residual confounding and so plots are not separated by DGM

Figure 8 contains the average calibration of each method when there was no residual correlation present. For the higher marginal incidence scenario (HN) all the methods had near perfect average calibration (slight deviations for the dual‐outcome method). For the lower marginal incidence scenario (LN) there was greater differences in the performance of each method. We found that the product method and Frank copula methods had the best average calibration across sample sizes, however at *N* = 5000 the dual‐outcome and msm models also performed well. The Clayton and Gumbel copulas and both frailty models over predicted risk at all sample sizes.

## 4 CLINICAL EXAMPLE

### 4.1 Aims and setting

The aim of this clinical example was to assess the performance of each of the methods outlined in Section 2 in a real clinical setting. We considered the prediction of the 10‐year risk of CVD and T2D developing together. The impact of multimorbidity on healthcare systems and patient outcomes has been well documented,^27^, ^28^, ^29^, ^30^, ^31^, ^32^, ^33^, ^34^ as well as an increased economic burden specifically for those with CVD and T2D,^83^, ^84^ and increased levels of mortality.^85^, ^86^ Knowing the risk of developing both of these conditions for specific groups of individuals in the population would enable health care providers to optimize resource allocation.

### 4.2 Methods

#### 4.2.1 Data source

Data from the Clinical Practice Research Datalink (CPRD), linked to admitted patient care data from Hospital Episode Statistics (HES) and death data from the Office for National Statistics (ONS), was used to build these models. CPRD GOLD and CPRD Aurum are primary care datasets containing data from general practices with the Vision and EMIS Web computer systems, respectively.^87^ CPRD Aurum was used is this study, which covers practices in England and Northern Ireland, with >39 million historical patients, and > 13 million currently registered. It is representative of the English population in terms of age, gender, geographical spread and deprivation (as of 2019).^88^

#### 4.2.2 Outcomes and predictors

We extracted a cohort including all patients that had at least 1 day of follow up in the database aged >65 after 1 January 2000, and at least 1 year up to standard registration prior to this point. Start of follow up was defined as the maximum of date turned age 65, 1 January 2000, and date of 1 year of up to standard registration in the database. End of follow up was defined as the minimum of date of death, transferred out of practice, or last data collection for practice. Individuals were then excluded if they had a history of CVD or T2D event prior to their start of follow up. CVD and T2D events were identified through the CPRD, HES, and ONS data sources. CVD was defined as a composite event consisting of heart failure, myocardial infarction, coronary heart disease, stroke, and transient ischaemic attack. We considered the following predictors at start of follow up for each individual: age, gender, smoking status (never, ex‐smoker, current smoker), systolic blood pressure (SBP), cholesterol/high density lipoprotein (chol/HDL) ratio, index of multiple deprivation (IMD), body mass index (BMI), and ethnicity (Black, Chinese and other, Mixed race, South Asian, White). We included all variables included in the SCORE risk prediction model (used for CVD risk assessment across Europe), plus any test data variables used as predictors in QRISK3 (used for CVD risk assessment in England and Wales). Predictor variables were identified through CPRD only. Code lists for all variables and algorithms for extracting test data are provided on GitHub.^76^ Operational definitions for extracting all variables and details on the code‐lists are given in supporting information file 1.

#### 4.2.3 Data preparation

*N* = 2 074 323 individuals met the inclusion/exclusion criteria and were included in the cohort. There were missing data on Smoking status, SBP, chol/HDL ratio, BMI, IMD, and ethnicity. We wanted to focus on what happened without missing data, we therefore created a pseudo “complete” case dataset by imputing missing values using a single stochastic imputation, obtained through a single multiple imputation chain.^89^ In practice we recommend implementing a full multiple imputation. $N_{\mathrm{dev}}=100\;000$ and $N_{\mathrm{val}}=100\;000$ individuals were then selected at random for the development and validation cohorts. More details on the imputation process, including convergence and density plots for all imputed variables, are provided in supporting information file 1.

#### 4.2.4 Data analysis and performance measures

Let $T_{\mathrm{CVD},T2D}$ be the time until both CVD and T2D have occurred. Models were developed in the development cohort to predict the 10‐year risk, $P_{C\mathrm{VD},T2D}=P\left(T_{\mathrm{CVD},T2D}<3652.25|X\right)$, using each of the methods outlined in Section 2. This was done using the survival package^90^ (product method and dual‐outcome method), GJRM^52^ (copula models), rstan^91^ (frailty models) and mstate^63^, ^64^ (multistate model). The frailty models were fit using Bayesian statistical inference and Monte Carlo Markov chains, assuming a Weibull marginal baseline hazard for each outcome. Prior distributions, the distributions from which initial values were drawn from and convergence plots are provided in supporting information file 1. For the Clayton and Gumbel copula models, we tested rotations of the copula of 90°, 180°, and 270° (rotations were not possible with the Frank copula). Calibration of each was assessed visually and the best fitting copula was used in the final analysis.

Predicted risks ${\hat{P}}_{\mathrm{CVD},T2D}$ were then generated for each individual in the validation cohort. We assessed calibration using graphical calibration curves.^92^ To do this, the complementary loglog transformation of ${\hat{P}}_{\mathrm{CVD},T2D}$, ${\hat{\text{CLOGP}}}_{\mathrm{CVD},T2D}=\log\left(-\log\left(1-P_{\mathrm{CVD},T2D}\right)\right)$, was used as the sole predictor in a cox proportional hazards model, predicting $T_{\mathrm{CVD},T2D}$:

$$h_{\mathrm{CVD},T2D}\left(t\right)=h_{0}\left(t\right)*\left(\mathrm{rcs}\left({\hat{\text{CLOGP}}}_{\mathrm{CVD},T2D}\right)\right),$$

where $h_{\mathrm{CVD},T2D}\left(t\right)$ is the hazard function for $T_{\mathrm{CVD},T2D}$, and rcs denotes restricted cubic splines (5 knots) on the predictor variable. Observed risks were then estimated by estimating a baseline hazard function for this model and calculating fitted values for each individual in the validation cohort using this model.

This approach places an assumption of proportional hazards on the outcome with respect to the complementary log‐log transformation of the predicted risks, which may not be valid. We allow some deviation from this assumption by introducing cubic splines. We therefore also split the validation cohort into deciles of predicted risk and calculated the average predicted risk, and a Kaplan‐Meier estimate of observed risk within each decile, to give a binned calibration plot.^93^ While this approach has its own limitations (categorization of a continuous variable resulting in loss of information), this is a nonparametric way of assessing calibration and provides an alternative assessment of calibration. Discrimination was assessed using Harrell's C.^80^

### 4.3 Results

**FIGURE 9:**
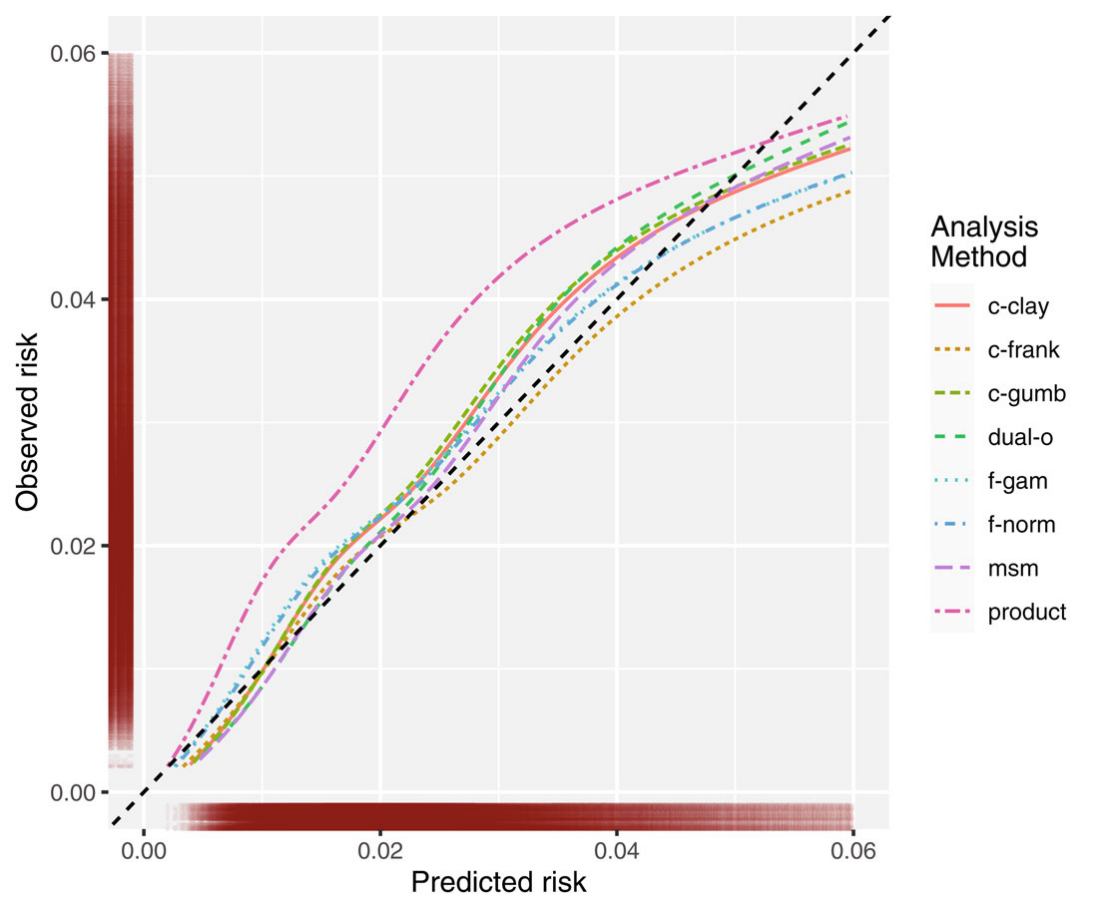
Graphical calibration curves of each method in the clinical example

Baseline data on development and validation cohorts are provided in supporting information file 2: Table S6.1. Figure 9 contains graphical calibration curves for each method. We have plotted over the majority of the density of the predicted risk, but not the full range, to allow a more granular comparison of the methods. See supporting information file 2: Figure S6.1 for a plot over the full range of predicted risk. The product method was the worst calibrated, often underpredicting risk. In comparison, all the other methods were well calibrated, although suffered from over prediction at higher predicted risks. Of the remaining methods, the Frank copula had the best calibration up to a predicted risk of 0.04 but suffered the most from the over prediction of risk at the higher risk values. The msm had the next best calibration. The dual‐outcome method had poor calibration between predicted risk of 0.03 to 0.04, but had very good calibration below 0.03, and suffered the least from over prediction at the higher end of predicted risks. Moderate calibration assessed by observed (Kaplan‐Meier) vs predicted risk within deciles of predicted risk is presented in supporting information file 2 (Figure S6.2). This again showcases that the dual‐outcome method was the least affected by the extreme values, with the highest risk decile being perfectly calibrated. In these plots, dual‐outcome and msm were also the only methods where the observed risks increased for each decile of each predicted risk. This highlights a level of miscalibration not picked up by the graphical calibration curves. Harrel's C‐statistic of all methods was the same (0.69).

## 5 DISCUSSION

### 5.1 Summary from the simulation

The product method had very poor calibration in the presence of residual correlation and had the worst average calibration of all the analysis methods across all scenarios. We therefore do not recommend using this approach in practice. The msm and dual‐outcome methods were the most robust to model misspecification as they were the only methods to have similar levels of performance across all the DGMs. This is likely because they do not make parametric assumptions on the distribution of the residual correlation, unlike all the other methods. For larger sample sizes they were also the best calibrated methods, meaning if the sample size is sufficient, these would be the most appropriate methods to use in practice. On the contrary, these methods were the most prone to miscalibration issues at small sample sizes. They also had the highest levels of variability in calibration across simulation iterations (although the differences between each method became negligible at bigger sample sizes). As the marginal incidence of each outcome increased the performance of all the methods became more similar (although the product method and Gumbel copula still had the worst calibration across all the scenarios). The dual‐outcome and msm approaches performed well even at small sample sizes in this context, the higher marginal incidences overcoming their power limitations.

### 5.2 Summary from the clinical example

Considering the very poor calibration of Frank copula at the higher range of predicted risk, dual‐outcome and msm had the best calibration. Alike to the simulation, the discrimination of all methods was the same. From a computational perspective (which was only an issue due to the large sample size of 100 000 used in this clinical example) the dual‐outcome and product method models were fit quickly, whereas the copula models took a couple of hours, and the frailty models both took approximately a week to run, with each MCMC being run in parallel. It is possible this runtime could be reduced by better choice of priors and starting values. The msm model also took over a week to run, largely driven by the time it takes to generate predicted risks for each individual in the validation cohort. The dual‐outcome and product methods could therefore be easily extended to model more predictor parameters and a higher number of individuals, which is not the case for the other methods.

### 5.3 Overall discussion

**Aim 1, to measure the extent of the miscalibration in predicting the risk of both‐of‐two survival outcomes using the product method, when there is residual correlation in the outcomes**: If predicting the co‐occurrence of multiple time‐to‐event outcomes is of interest, then a technique that models the residual correlation is essential to obtain a well calibrated estimate of risk in the presence of residual confounding. This was highlighted by the poor calibration of the product method throughout the simulation in this study. Furthermore, the product method was found to be miscalibrated in a real clinical setting predicting the risk of CVD and T2D both occuring. This is an important finding because the product method is only biased if the conditional independence assumption does not hold. One may therefore argue that when adjusting for major risk factors with established biological mechanisms, that the bias of the product method may be negligible. However, we found that when adjusting for 8 major cardiovascular risk factors, which are common risk factors for T2D too, there was still a significant level of miscalibration and under prediction of the risk.

**Aim 2, to compare the performance of available methods for predicting the risk of both‐of‐two survival outcomes**: To estimate the risk of both‐of‐two survival outcomes in the presence of residual confounding we recommend the dual‐outcome approach. This method, along with the msm, was the most robust to model misspecification, had good calibration across a wide range of scenarios and performed well in the clinical example. This method is also the most practical as it can be implemented using standard survival analysis techniques. This means nonlinear modeling of predictors,^94^, ^95^, ^96^, ^97^, ^98^, ^99^ accounting for competing risks^40^, ^41^, ^42^, ^43^, ^44^ and implementation of variable selection^100^, ^101^ can be easily achieved using existing methodology. How to approach these topics within the framework of the other modeling approaches is currently unclear. If discrimination is the most important performance metric on which a model is being assessed, our simulation indicates all methods are equally valid options. However, we still recommend the dual‐outcome approach given its robustness to model misspecification in terms of calibration.

However, there are drawbacks to this approach which must be made clear. First, this method suffered from a drop in calibration performance at small sample sizes driven by overfitting. Here, the copula (in particular Clayton and Frank) and frailty models sometimes gave better calibration, yet which model had the best calibration was dependent on the DGM. This highlights that at small sample sizes it becomes more important to understand the underlying data structure and avoid model misspecification. On the contrary, while the calibration of the dual‐outcome method was poor at small sample sizes, it was consistent across all DGMs, highlighting the method's robustness to model misspecification. Poor calibration induced by overfitting can be mediated by reducing the number of predictors and ensuring sample size criteria are met,^77^ or applying shrinkage and penalisation techniques. When developing a model on a small development dataset this may be an alternative approach, however would come at a cost to the discrimination of the model, which must therefore be weighed up against the risks of misspecifying the model if using a copula or frailty model.

Another drawback of the dual‐outcome approach is that it does not give a direct measure of the level of association between the two outcomes. On the contrary, all of the other methods provide a quantifiable assessment of the level of dependence between the two outcomes: the estimated distribution parameter of the copula models (θ); the variance of the random effect $\omega_{i}$ in the frailty models; and the estimated increase in the hazard rate after conditions have been developed in the msm ($\lambda_{A,B}$ and $\lambda_{B,A}$). The dual‐outcome method likely has the best performance in our study as it directly targets the estimand that we are interested in, whereas the other approaches are restricted into parametric structures designed to estimate the level of dependence, which may not be appropriate. It is therefore important to establish the primary research question and potential uses of the model when choosing the modeling approach. While the dual‐outcome model is most suitable for estimating $P\left(T_{A}\leq t,T_{B}\leq t|X\right)$, if any interest lies in estimating the dependence structure other models must be considered at the potential cost of some predictive performance.

There are a number of other points of note to raise about each of the methods. Unlike all the other methods, the dual‐outcome model does not also allow estimation of the marginal risks within a common framework. Arguably this is not an issue, as developing separate survival models to estimate these would be no more complex than implementing the other proposed approaches. The frailty approach has an added benefit that it can be fit to datasets with individuals who are missing either outcome A or B (but not both). The correlation in the observed data can be used to recover some of the missing information, which is a more common scenario where multivariate methods may be used. It is unclear whether this would be preferable over using an “impute then model” type approach, or even running a complete case analysis, and further work is needed here.

Table 2 contains a summary of the main conclusions from the study. The conclusions are categorized based on the three major aspects of the simulation that were varied (outlined in Section 3.2.4) and an “other” category.

**Limitations**: We focused only on two outcomes to serve as initial work in this space; however, all the methods considered could be used to predict the risk of more than two time‐to‐event outcomes. Further research will be needed to explore their behavior as the number of outcomes increases. We also assumed a common censoring mechanism for both outcomes, although all the methods compared in this study can be applied when the two outcomes have different censoring mechanisms, and we can hypothesize no reason why this would affect performance. Despite this, it may be worthwhile exploring this in future work. As is the case for all simulations, it is possible that the data on which our simulation was based is not representative of real clinical data on which the methods would be used in practice. We tried to alleviate this by considering a range of DGMs, 6 in total, and made each modeling approaches ability to perform across this range of DGMs a key aspect of the simulation and how we interpreted the results. We also considered a range of scenarios, of which the primary one (scenario LL), was matched to our clinical example. Furthermore, we compared the performance of each method in a real clinical example, and we were able to confirm the fallibility of the product method in this setting. The strong calibration of both the dual‐outcome method and msm method in the clinical example further validates the findings from the simulation. Finally, one of the main findings was the relative impact of overfitting on the dual‐outcome approach compared to the others at small sample sizes. However, we did not implement any shrinkage or penalisation techniques, which may alleviate this issue to some extent. Given that software is widely available to apply these techniques on standard survival models, but less common place for msm's, copulas and frailty models, this is another potential advantage of the dual‐outcome approach. Further research is needed to understand the performance of all these methods when penalisation and shrinkage is applied.

**TABLE 2:** Summary of main conclusions from the study

Simulation aspect	Conclusion
Changing DGM (evaluating robustness to model misspecification)	The dual‐outcome and msm approaches were the most robust to model misspecification. They had consistent performance across all DGMs, whereas the frailty and copula models were more sensitive to the choice of DGM.
Increasing the level of residual correlation	Increasing the level of residual correlation had no impact on model performance of the methods relative to each other (excluding the product method). Dual‐outcome and msm where the most robust to model misspecification for the lower and higher levels of residual correlation.
Increasing the sample size or marginal risks	The dual‐outcome and msm were the most prone to overfitting at lower sample sizes and when marginal risks were lower. Even when impacted by overfitting, the dual‐outcome and msm approaches were still the most robust to model misspecification
Other	Discrimination of all methods was very similar. In the clinical example, the product method resulted in a miscalibrated estimate of the risk of CVD and T2D, despite conditioning on 8 major risk factors. This suggests the conditional independence assumption may not hold in practice and motivates the use of the methods considered in this study.

## 6 CONCLUSIONS

This is the first study to compare modeling techniques for the prediction of the risk of two survival outcomes both occurring in the presence of residual confounding. In the clinical example, the product method resulted in a miscalibrated estimate of the risk of CVD and T2D, indicating the conditional independence assumption was violated despite conditioning on 8 major risk factors. This motivates the need for techniques which appropriately model the residual dependence. In the simulation, all models resulted in similar levels of discrimination, however variable performance was found with respect to discrimination. The dual‐outcome and msm methods were the most robust to model misspecification. Although these methods were also the most prone to overfitting (this was observed at minimum sample sizes according to existing criteria), this is an issue that can be solved by reducing the number of predictor variables or recruiting more individuals. On the contrary, the poor calibration of the other methods induced by model misspecification cannot be dealt with. When sample sizes are very large (such as in the clinical example), we recommend the dual‐outcome approach alone, due to its comparative performance to the other methods alongside a substantially lower computational time.

## AUTHOR CONTRIBUTIONS

Alexander Pate and Glen P. Martin conceived and designed the study in discussion with Matthew Sperrin, Richard D. Riley, Iain Buchan, and Jamie C. Sergeant. Alexander Pate conducted the analysis and interpreted the results in discussion with all authors. Alexander Pate wrote the initial draft of the article with support from Glen P. Martin, which was then critically reviewed for important intellectual content by all authors. All authors have approved the final version of the article.

## FUNDING INFORMATION

This work was supported by funding from the MRC‐NIHR Methodology Research Programme [grant number: MR/T025085/1].

## CONFLICT OF INTEREST STATEMENT

No competing interest.

## ETHICS STATEMENT

Access to Clinical Practice Research Datalink data is supported by ISAC protocol 20_000102.

## Supporting information


**Supporting information file 1.** Supplementary material on methodology.

**Supporting information file 2.** Supplementary tables and figures.

## Data Availability

Reusable code is available from our GitHub public repository.^76^ Data for the clinical example cannot be shared and must be obtained through an application to the Clinical Practice Research Datalink. The simulation was implemented in R version 4.1.2,^32^ and rstudio^102^ using the following packages: mstate,^63^, ^64^ GJRM,^52^, ^53^ rstan,^78^ cubature,^103^ survAUC,^104^ mfp,^98^ dplyr,^105^ Hmisc,^106^ rms,^107^ ggplot2,^108^ Cairo,^109^ Desctools,^110^ ggpubr,^111^ knitr,^112^ reshape2,^113^ mice,^89^ gems,^114^ and simsurv.^115^ Analysis were run on the computation shared facility at University of Manchester.
