# Supplementary material for: Developing prediction models to estimate the risk of two survival outcomes both occurring: A comparison of techniques
Source: Stat Med. 2023 May 23;42(18):3184–207. doi: 10.1002/sim.9771 (PMC11155421; doi:10.1002/sim.9771)

Appendix S1: Supplementary information on methodology

Developing prediction models to estimate the risk of two survival outcomes both occurring: a comparison of techniques

Table of Contents

[SM1 Proof of equation from section 2.4 3](#_Toc126234055)

[SM2 Distributional assumptions for the dual-outcome approach 5](#_Toc126234056)

[SM3 Process for calculating the true joint risk, $PA\leq t, B\leq tX$, and marginal risks, $PA\leq tX$ and $PB\leq tX$, for a given individual for each DGM 6](#_Toc126234057)

[3.1 Calculating the true joint risk for a given set of input parameters for each DGM 6](#_Toc126234058)

[3.1.1 True joint risk for DGM-1: multistate model 6](#_Toc126234059)

[3.1.2 True risk for copula DGM 7](#_Toc126234060)

[3.1.3 True risk for frailty DGM 7](#_Toc126234061)

[SM4 Process for calculating the mean joint risks under various assumptions ($jrind$*,* $jrpred$ and $jrtrue$) for each DGM, and how these were used to shape simulation scenarios 8](#_Toc126234062)

[4.1 Calculating $jrind$ 8](#_Toc126234063)

[4.2 Calculating $jrpred$ 8](#_Toc126234064)

[4.3 Calculating $jrtrue$ 8](#_Toc126234065)

[4.4 How we targeted simulation scenarios to match clinical data 8](#_Toc126234066)

[SM5 Input parameters used to generate data in simulation 9](#_Toc126234067)

[SM6 Process for calculating median calibration curves and 5 – 95 percentile range in calibration curves 10](#_Toc126234068)

[SM7 Operational definitions for extracting variables 11](#_Toc126234069)

[SM8 Code lists 12](#_Toc126234070)

[8.1 Code lists used for primary care extraction 12](#_Toc126234071)

[8.2 Code lists used for HES/ONS extraction 14](#_Toc126234072)

[SM9 Multiple imputation methodology and evaluation of imputed data 15](#_Toc126234073)

[9.1 Methods 15](#_Toc126234074)

[9.2 Evaluation of imputed data 15](#_Toc126234075)

[9.2.1 Imputation of female cohort 15](#_Toc126234076)

[9.2.2 Imputation of male cohort 21](#_Toc126234077)

[SM10 Details of normal and gamma frailty models, priors, initial values and convergence plots 26](#_Toc126234078)

[10.1 Normal frailty model 26](#_Toc126234079)

[10.1.1 Definition of model and priors 26](#_Toc126234080)

[10.1.2 Initial values 26](#_Toc126234081)

[10.1.3 Convergence plots 28](#_Toc126234082)

[10.2 Gamma frailty model 29](#_Toc126234083)

[10.2.1 Definition of model and priors 29](#_Toc126234084)

[10.2.2 Initial values 30](#_Toc126234085)

[10.2.3 Convergence plots 31](#_Toc126234086)

# Proof of equation from section 2.4

We proposed that the joint risk can be estimated using the following equation:

$$P\left( A\leq t, B\leq t | X \right)=1-P\left( A>t | X \right)-P\left( B>t | X \right)+C_{\theta}\left\{ P\left( A>t | X \right), P\left( B>t | X \right) \right\}$$

This result follows from the fact that:

$P\left( A\leq t B\leq t | X \right)+P\left( A>t, B\leq t | X \right)+P\left( A\leq t, B>t | X \right)+P\left( A>t, B>t | X \right)=1$ ⬄

$$P\left( A\leq t, B\leq t | X \right)=1-P\left( A>t, B\leq t | X \right)-P\left( A\leq t, B>t | X \right)-P\left( A>t, B>t | X \right)$$

and that:

$$P\left( A>t, B\leq t | X \right)=P\left( A>t | X \right)-P\left( A>t, B>t | X \right)$$

$$P\left( A\leq t, B>t | X \right)=P\left( B>t | X \right)-P\left( A>t, B>t | X \right)$$

Giving:

$$P\left( A\leq t, B\leq t | X \right)=1-P\left( A>t | X \right)-P\left( B>t | X \right)+P\left( A>t, B>t | X \right)$$

$$P\left( A\leq t, B\leq t | X \right)=1-P\left( A>t | X \right)-P\left( B>t | X \right)+C_{\theta}\left\{ P\left( A>t | X \right), P\left( B>t | X \right) \right\}$$

# Distributional assumptions for the dual-outcome approach

The survival function for the dual outcome $AB$ can be written as

$S_{AB}\left( t|X \right)=P\left( AB>t|X \right)=P\left( A>t,B<t|X \right)+P\left( A<t,B>t|X \right)+P\left( A>t, B>t|X \right)$.

Lets consider the simplest case, where there is no residual correlation between $A$ and $B$ given $X$ (i.e. a scenario where the product method is appropriate). Then we have:

$$S_{AB}\left( t|X \right)=P\left( A>t|X \right)*P\left( B<t|X \right)+P\left( A<t|X \right)*P\left( B>t|X \right)+P\left( A>t|X \right)*P\left( B>t|X \right)$$

$$=P\left( A>t|X \right)*\left( 1-P\left( B>t|X \right) \right)+P\left( B>t|X \right)*\left( 1-P\left( A>t|X \right) \right)+P\left( A>t|X \right)*P\left( B>t|X \right)$$

$$=P\left( A>t|X \right)*\left( 1-P\left( B>t|X \right) \right)+P\left( B>t|X \right)*\left( 1-P\left( A>t|X \right) \right)+P\left( A>t|X \right)*P\left( B>t|X \right)$$

$$=P\left( A>t|X \right)+P\left( B>t|X \right)-P\left( A>t|X \right)*P\left( B>t|X \right)$$

$$=S_{A}\left( t \right)+S_{B}\left( t \right)-S_{A}\left( t \right)*S_{B}\left( t \right)$$

$$=\exp\left( -H_{A}\left( t|X \right) \right)+\exp\left( -H_{B}\left( t|X \right) \right)-\exp\left( -H_{A}\left( t|X \right) \right)*\exp\left( -H_{B}\left( t|X \right) \right)$$

For proportional hazards to hold, the predictor variables in $X$ must have a multiplicative effect on the log of this function. For the accelerated failure time model, predictor variables in $X$ must have a multiplicative effect on the mean failure times resulting from this distribution. Neither seem particularly likely. Suppose that proportional hazards holds for both $A$ and $B$. Then $S_{A}\left( t|X \right)=\exp\left( -H_{0,A}\exp\left( X.\beta_{A} \right) \right)$ and $S_{B}\left( t|X \right)=\exp\left( -H_{0,B}\exp\left( X.\beta_{B} \right) \right)$. We then have:

$$S_{AB}\left( t|X \right)=\exp\left( -H_{0,A}\exp\left( X.\beta_{A} \right) \right)+\exp\left( -H_{0,B}\exp\left( X.\beta_{B} \right) \right)-\exp\left( -H_{0,A}\exp\left( X.\beta_{A} \right)-H_{0,B}\exp\left( X.\beta_{B} \right) \right)$$

It is unclear how to write this in the form $S_{AB}\left( t|X \right)=\exp\left( -H_{0,AB}\exp\left( X.\beta_{AB} \right) \right)$.

We therefore propose that any distributional assumptions on the dual-outcome $AB$ are stronger than placing them on either $A$ or $B$ in isolation. From a non-statistical point of view, this makes sense, given that $A$ and $B$ may both interact with each other and $X$ in a number of different ways, and there is no reason to assume the dual-outcome $AB$ would have the desired properties of the chosen model.

The key question of this simulation, is whether violation of these assumptions causes greater levels of miscalibration than misspecification of the other modelling approaches, across a range of data generating mechanisms.

# Process for calculating the true joint risk, $P\left( A\leq t, B\leq t | X \right)$, and marginal risks, $P\left( A\leq t | X \right)$ and $P\left( B\leq t | X \right)$, for a given individual for each DGM

The true joint risk for a given individual under a particular DGM was an important quantity in this simulation. The calibration plots were produced by regressing the true joint risk (calculated using the processes outlined here) on the predicted risk. The true joint risks and marginal risks were also used to calculate ${jr}_{ind}$, ${jr}_{pred}$ and ${jr}_{true}$, the mean joint risk across the population under various assumptions, which were used to create the different scenario sin the simulation (see section SM4). The following functions are written and implemented to calculate the true joint risks and marginal risks in the program “*p0_sim_calculate_input_parameters_2cont.R*”, available on GitHub.^1^

## Calculating the true joint risk for a given set of input parameters for each DGM

This section details how from a given set of input parameters, we can calculate the true value of $P\left( A\leq t, B\leq t | X \right)$ for each DGM.

### True joint risk for DGM-1: multistate model

For a multistate model

$$P\left( A\leq t, B\leq t | X \right)=P\left( A<B\leq t|X \right)+P\left( B<A\leq t|X \right)$$

This is the probability of being in state $A+B$ at time $t$, which is equal to the probability of being in state $A+B$ at time $t$ via state $A$, plus the probability of being in state $A+B$ at time $t$ via state $B$. Following the theory in Putter et al.,^2^:

$$P\left( A\leq B\leq t|X \right)=\int_{0}^{t} \lambda_{0,A}(r)S_{0}\left( r \right)P_{A,AB,r}\left( r,t \right)dr$$

Where:

$\lambda_{0,A}(r)$ is the cause-specific hazard for transition $0 -> A$,

$S_{0}\left( r \right)$ is probability of staying in state $0$ until time $r$,

$P_{A,AB,r}\left( r,t \right)$ is the probability of moving into state $B$ between time $r$ and $t$, after entering state $A$ at time $r$.

The subscript $X$ has been dropped in the above equation for brevity, but all these functions are conditional on the set of patient characteristics $X$ at baseline. All these functions can be calculated directly from the cause-specific hazards detailed in section 3.1.2.1 of the manuscript.

Similarly,

$$P\left( B\leq A\leq t|X \right)=\int_{0}^{t} \lambda_{0,B}(r)S_{0}\left( r \right)P_{B,AB,r}\left( r,t \right)dr$$

The marginal probability of $P\left( A\leq t | X \right)$ is the sum of the probability of being in state $A+B$ at time $t$, $P\left( A\leq t, B\leq t | X \right)$, plus the probability of being in state $A$ at time $t$, $P\left( A\leq t, B\geq t | X \right)$.

$$P\left( A\leq t, B\geq t | X \right)=\int_{0}^{t} \lambda_{0,A}(r)S_{0}\left( r \right)P_{A,A,r}\left( r,t \right)dr$$

Where:

$\lambda_{0,A}(r)$ and $S_{0}\left( r \right)$ are defined as above, and $P_{A,A,r}\left( r,t \right)$ is the probability of staying in state $A$ between time $r$ and $t$, after entering state $A$ at time $r$. $P\left( A\leq t | X \right)$ can then be calculated as:

$$P\left( A\leq t | X \right)=P\left( A\leq t, B\leq t | X \right)+P\left( A\leq t, B\geq t | X \right)$$

And similarly:

$$P\left( B\leq t | X \right)=P\left( A\leq t, B\leq t | X \right)+P\left( A\geq t, B\leq t | X \right)$$

### True risk for copula DGM

For the copula model, the true joint risk is calculated as:

$$P\left( A\leq t_{A}, B\leq t_{B} | X \right)=1-P\left( A>t_{A} | X \right)-P\left( B>t_{B} | X \right)+C_{\theta}\left\{ P\left( A>t_{A} | X \right), P\left( B>t_{B} | X \right) \right\}$$

The marginal survival probabilities $P\left( A>t_{A} | X \right)$ and $P\left( B>t_{B} | X \right)$ can be calculated from the marginal distributions used at the data generation stage (section 3.1.2.2), and $C_{\theta}$ is the copula defined at the data generation stage.

### True risk for frailty DGM

For a frailty model, the true joint risk is calculated by integrating out the frailty term:

$$P\left( A\leq t_{A}, B\leq t_{B} | X_{i} \right)=\int P\left( A\leq t_{A} | X_{i},\omega\right)*P\left( B\leq t_{B} | X_{i},\omega\right)f_{\omega}(\omega)d\omega$$

The marginal survival probabilities given $\omega$, $P\left( A\leq t_{A} | X_{i},\omega\right)$ and $P\left( B\leq t_{B} | X_{i},\omega\right)$, can be calculated from the marginal distributions used at the data generation stage (section 3.1.2.3), $f_{\omega}\left( \omega\right)$ is the probability density function for the distribution defined at the data generation stage.

# Process for calculating the mean joint risks under various assumptions (${jr}_{ind}$*,* ${jr}_{pred}$ and ${jr}_{true}$) for each DGM, and how these were used to shape simulation scenarios

${jr}_{ind}$ is the mean joint risk in population assuming complete independence (no conditioning on predictors). ${jr}_{pred}$ is the mean joint risk in population assuming independence after conditioning on available predictors. ${jr}_{true}$ is the true mean joint risk in population. We developed scenarios based around targeting these values to be at a certain level. Note that in the below equations, $P\left( A\leq t_{A} | X \right)$, $P\left( B\leq t_{B} | X \right)$ and $P\left( A\leq t_{A}, B\leq t_{B} | X \right)$ are specific to each DGM. These were calculated using the processes outlined in section SM3 of this Appendix. The following functions are written and implemented to calculate ${jr}_{ind}$*,* ${jr}_{pred}$ and ${jr}_{true}$ in the program “*p0_sim_calculate_input_parameters_2cont.R*”, available on GitHub.^1^

## Calculating ${jr}_{ind}$

${jr}_{ind}$ was the product of the marginal risks, each integrated over the distribution of $X_{1}$ and $X_{2}.$

$${jr}_{ind}=\left( \int P\left( A\leq t | X \right)*f_{X_{1}}\left( X_{1} \right)*f_{X_{2}}\left( X_{2} \right) dX_{1}dX_{2} \right)*\left( \int P\left( B\leq t | X \right)*f_{X_{1}}\left( X_{1} \right)*f_{X_{2}}\left( X_{2} \right) dX_{1}dX_{2} \right)$$

## Calculating ${jr}_{pred}$

${jr}_{pred}$ is the product of the marginal risks ecah adjusted for $X$, integrated over the distribution of $X_{1}$ and $X_{2}.$

$${jr}_{pred}=\int P\left( A\leq t_{A} | X \right)*P\left( B\leq t_{B} | X \right)*f_{X_{1}}\left( X_{1} \right)*f_{X_{2}}\left( X_{2} \right) dX_{1}dX_{2}$$

## Calculating ${jr}_{true}$

${jr}_{true}$ is the true joint risk, integrated over the distribution of $X_{1}$ and $X_{2}.$

$${jr}_{pred\_res}=\int P\left( A\leq t_{A}, B\leq t_{B} | X \right)*f_{X_{1}}\left( X_{1} \right)*f_{X_{2}}\left( X_{2} \right) dX_{1}dX_{2}$$

## How we targeted simulation scenarios to match clinical data

We were particularly interested in ${{jr}_{true}}/{{jr}_{pred}}$*,* which is a measure of the amount of residual correlation after having adjusted for predictor variables. ${jr}_{pred}$ can be interpreted as the risk that would be estimated if using the product method to analyse the data, whereas ${jr}_{true}$ is the joint risk that would be estimated when appropriately modelling the dependence between the two outcomes. We allowed this ratio to be 1 (no residual correlation), 1.2 (high residual correlation) and 1.5 (high residual correlation).

# Input parameters used to generate data in simulation

*Table SM1: Input parameters for each scenario*

| **Scenario LN** | | | | | | | | | | | |
| --- | --- | --- | --- | --- | --- | --- | --- | --- | --- | --- | --- |
| **DGM** | $\boldsymbol{\lambda}_{\boldsymbol{A}}$ | | $\boldsymbol{\lambda}_{\boldsymbol{B}}$ | | $\boldsymbol{\beta}_{\boldsymbol{A}}$ | | $\boldsymbol{\beta}_{\boldsymbol{B}}$ | | **Frailty parameter 1** | | **Frailty parameter 2** |
| Clayton | 115 | | 120 | | (0.25, 0.6) | | (0.73, 0.15) | | $\theta$ = 0.000001 | | NA |
| **Scenario LL** | | | | | | | | | | | |
| **DGM** | | $\boldsymbol{\lambda}_{\boldsymbol{A}}$ | | $\boldsymbol{\lambda}_{\boldsymbol{B}}$ | | $\boldsymbol{\beta}_{\boldsymbol{A}}$ | | $\boldsymbol{\beta}_{\boldsymbol{B}}$ | | **Frailty parameter 1** | **Frailty parameter 2** |
| MSM | | 118 | | 123 | | (0.25, 0.6) | | (0.73, 0.15) | | $\lambda_{A,B}$ = 0.77 | $\lambda_{B,A}$ = 0.77 |
| Clayton | | 115 | | 120 | | (0.25, 0.6) | | (0.73, 0.15) | | $\theta$ = 0.28 | NA |
| Gumbel | | 115 | | 120 | | (0.25, 0.6) | | (0.73, 0.15) | | $\theta$ = 1.03 | NA |
| Frank | | 115 | | 120 | | (0.25, 0.6) | | (0.73, 0.15) | | $\theta$ = 0.65 | NA |
| Normal | | 126 | | 131 | | (0.25, 0.6) | | (0.73, 0.15) | | $\sigma$ = 0.5 | NA |
| Gamma | | 112 | | 117 | | (0.28, 0.6) | | (0.73, 0.15) | | $\beta$ = 0.27 | NA |
| **Scenario LH** | | | | | | | | | | | |
| **DGM** | $\boldsymbol{\lambda}_{\boldsymbol{A}}$ | | $\boldsymbol{\lambda}_{\boldsymbol{B}}$ | | $\boldsymbol{\beta}_{\boldsymbol{A}}$ | | $\boldsymbol{\beta}_{\boldsymbol{B}}$ | | **Frailty parameter 1** | | **Frailty parameter 2** |
| MSM | 122 | | 126 | | (0.25, 0.6) | | (0.73, 0.15) | | $\lambda_{A,B}$ = 0.58 | | $\lambda_{B,A}$ = 0.58 |
| Clayton | 115 | | 120 | | (0.25, 0.6) | | (0.73, 0.15) | | $\theta$ = 0.69 | | NA |
| Gumbel | 115 | | 120 | | (0.25, 0.6) | | (0.73, 0.15) | | $\theta$ = 1.065 | | NA |
| Frank | 115 | | 120 | | (0.25, 0.6) | | (0.73, 0.15) | | $\theta$ = 1.37 | | NA |
| Normal | 144 | | 149 | | (0.25, 0.6) | | (0.73, 0.15) | | $\sigma$ = 0.77 | | NA |
| Gamma | 107 | | 112 | | (0.25, 0.6) | | (0.73, 0.15) | | $\beta$ = 0.69 | | NA |
| **Scenario HN** | | | | | | | | | | | |
|  | $\boldsymbol{\lambda}_{\boldsymbol{A}}$ | | $\boldsymbol{\lambda}_{\boldsymbol{B}}$ | | $\boldsymbol{\beta}_{\boldsymbol{A}}$ | | $\boldsymbol{\beta}_{\boldsymbol{B}}$ | | **Frailty parameter 1** | | **Frailty parameter 2** |
| Clayton | 35 | | 35 | | (0.3, 0.85) | | (0.9, 0.22) | | $\theta$ = 0.000001 | | NA |
| **Scenario HL** | | | | | | | | | | | |
| **DGM** | $\boldsymbol{\lambda}_{\boldsymbol{A}}$ | | $\boldsymbol{\lambda}_{\boldsymbol{B}}$ | | $\boldsymbol{\beta}_{\boldsymbol{A}}$ | | $\boldsymbol{\beta}_{\boldsymbol{B}}$ | | **Frailty parameter 1** | | **Frailty parameter 2** |
| MSM | 38 | | 38 | | (0.22, 0.8) | | (0.9, 0.2) | | $\lambda_{A,B}$ = 0.61 | | $\lambda_{B,A}$ = 0.61 |
| Clayton | 35 | | 35 | | (0.3, 0.85) | | (0.9, 0.22) | | $\theta$ = 0.55 | | NA |
| Gumbel | 35 | | 35 | | (0.3, 0.85) | | (0.9, 0.22) | | $\theta$ = 1.16 | | NA |
| Frank | 35 | | 35 | | (0.3, 0.85) | | (0.9, 0.22) | | $\theta$ = 1.52 | | NA |
| Normal | 39.5 | | 39.5 | | (0.3, 0.85) | | (0.9, 0.3) | | $\sigma$ = 0.75 | | NA |
| Gamma | 31 | | 31 | | (0.3, 0.85) | | (0.9, 0.3) | | $\beta$ = 0.45 | | NA |
| **Scenario HH** | | | | | | | | | | | |
| **DGM** | $\boldsymbol{\lambda}_{\boldsymbol{A}}$ | | $\boldsymbol{\lambda}_{\boldsymbol{B}}$ | | $\boldsymbol{\beta}_{\boldsymbol{A}}$ | | $\boldsymbol{\beta}_{\boldsymbol{B}}$ | | **Frailty parameter 1** | | **Frailty parameter 2** |
| MSM | 42 | | 43 | | (0.22, 0.7) | | (0.9, 0.15) | | $\lambda_{A,B}$ = 0.33 | | $\lambda_{B,A}$ = 0.33 |
| Clayton | 35 | | 35 | | (0.3, 0.85) | | (0.9, 0.22) | | $\theta$ = 2 | | NA |
| Gumbel | 35 | | 35 | | (0.3, 0.85) | | (0.9, 0.22) | | $\theta$ = 1.54 | | NA |
| Frank | 35 | | 35 | | (0.3, 0.85) | | (0.9, 0.22) | | $\theta$ = 4.2 | | NA |
| Normal | 50.5 | | 51.5 | | (0.4, 1) | | (1.1, 0.35) | | $\sigma$ = 1.38 | | NA |
| Gamma | 25 | | 25 | | (0.4, 1) | | (0.9, 0.3) | | $\beta$ = 1.45 | | NA |

The censoring event time was generated from an exponential distribution with rate $\lambda$ = 95, and log hazard ratios of $\beta$ = (0.1, 0.1). This resulted in approximately 5% of events prior to ten years being censored.

# Process for calculating median calibration curves and 5 – 95 percentile range in calibration curves

When presenting results of all the analysis methods, to give a sense of the The following process was carried out for each analysis method separately.

*Start loop. For* $i$ *in* $1:n.sim$*:*

1. Calculate predicted joint risk for each individual in the validation cohort

2. Calculate the true risk for each individual in the validation cohort, based off the input parameters from the data generating mechanism.

3. Fit a calibration model $mfp(risk_{true} \sim fp\left( risk_{pred} \right))$, where $rcs$ is a restricted cubic spline with 4 knots.

4. Use this model to generate expected true risks as a continuous function of the predicted risks, for a range of predicted risk values. We do not consider predicted risks outside the 1^st^ percentile and 99^th^ percentile of $P\left( A\leq t, B\leq t | X \right)$, as calculated using methods given in Appendix S1.

*End loop.*

5. We now have the expected true risk as a smooth function of predicted risk (calibration curves) for each run of the simulation, over the same set of predicted risk values. We plotted all calibration curves on the same graph. For each predicted risk in the sequence, we also calculated the median, 5^th^ and 95^th^ percentiles of the expected true risk from step 4.

6. Plot the median, 2.5^th^ and 97.5^th^ percentiles expected true risks as a function of the predicted risks, to produce the calibration plots.

# Operational definitions for extracting variables

The index date is defined as the start of follow up: maximum of date turned age 65, 1^st^ Jan 2000, and date of 1 year of up to standard registration in the database

For predictors which are comorbidities, we derived a variable which indicates whether an individual has a record of the comorbidity prior to their index date in their primary care record.

For predictors reliant on test data (BMI, SBP, cholesterol/HDL ratio, smoking status), we looked in the five years prior to the index date for an occurrence of the variable. Appropriate conversions were applied based on the unit of measurement recorded in the database. Extreme values were then removed. See Github page for full algorithms for each variable.

For outcomes, we derived the time until first occurrence or censoring, and a censoring indicator, in both the primary care, secondary care and ONS datasets. We then took the event as the time until the first of any of these to occur. We also derived presence of each condition at baseline, in order to apply our exclusion criteria.

# Code lists

All codelists are available on GitHub. Names of the final codelists used for data extraction, and details of where these were obtained is given in this section.

## Code lists used for primary care extraction

For this study, we opt to focus only on medical codes, and ignore prescription data. Table SM2 contains all the names of the code lists used for extraction of the primary care data and the source from which they were obtained. Code lists from source “AH” are available at the referenced Github page.^3,4^ Code lists from source “Cambridge mapped” were taken from the Cambridge primary care unit website,^5^ and then mapped by author TVS from CPRD GOLD to CPRD Aurum. Ethnicity code list was available at on the LSHTM Data Compass.^6,7^ The groupings for the medical codes were obtained from a Read code code list on another GitHub respository^8^, developed for another study.^9^ Some additional ethnicity codes were identified from this second code list, found in the CPRD Aurum code browser, and added to the code list. The smoking status code list was available on the LSHTM Data Compass.^10,11^ Self-generated code lists we developed by searching the CPRD Aurum code browser for *height*, *weight*, *body mass*, *systolic* and *cholesterol* respectively, and selecting the appropriate codes. Finally, we opted to use code lists for Psoriasis (as opposed to Psoriasis and eczema) and Rheumatoid arthritis (as opposed to rheumatoid arthritis and connective tissue disorders), due to the availability of code lists from source AH.

*Table SM2: Code lists file names and source*

|  | Variable | Code list name | Source |
| --- | --- | --- | --- |
| **Medical history** | | | |
| 1 | Alcohol problems | Alcohol_misuse | AH |
| 2 | Anorexia or bulimia | Eating_disorders | AH |
| 3 | Asthma (currently treated) | Asthma | AH |
| 4 | Atrial fibrillation | Atrial_fibrillation | AH |
| 5 | Anxiety or depression | Anxiety_disorders | AH |
| 5 | Anxiety or depression | Depression | AH |
| 6 | Blindness and low vision | Visual_impairment_and_blindness | AH |
| 7 | Bronchiectasis | Bronchiectasis | AH |
| 8 | Cancer Diagnosis in last five years | can_**** | AH |
| 9 | Chronic kidney disease | Chronic_kidney_disease | AH |
| 10 | Chronic Liver +Viral Hepatitis | Hepatic_failure | AH |
| 10 | Chronic Liver +Viral Hepatitis | Chronic_viral_hepatitis | AH |
| 11 | Chronic sinusitis | Chronic_sinusitis | AH |
| 12 | COPD | COPD | AH |
| 13 | Coronary heart disease | Coronary_heart_disease | AH |
| 14 | Dementia | Dementia | AH |
| 15 | Diabetes | Diabetes_mellitus_other_or_not_specified | AH |
| 15 | Diabetes | Diabetes_mellitus_type1 | AH |
| 15 | Diabetes | Diabetes_mellitus_type2 | AH |
| 16 | Diverticular disease of intestine | Diverticular_disease | AH |
| 17 | Epilepsy (currently treated) | Epilepsy | AH |
| 18 | Hearing loss | Hearing_loss | AH |
| 19 | Heart failure | Heart_failure | AH |
| 20 | Hypertension | Hypertension | AH |
| 21 | Inflammatory bowel disease | IBD160_mcid | TM |
| 22 | Irritable bowel syndrome | Irritable_bowel_syndrome | AH |
| 23 | Learning disability | Intellectual_disability | AH |
| 24 | Multiple sclerosis | Multiple_sclerosis | AH |
| 25 | Parkinson disease | Parkinsons_disease | AH |
| 26 | Peptic Ulcer Disease | PEP135_mcid | TM |
| 27 | Peripheral vascular disease | Peripheral_vascular_disease | AH |
| 28 | Prostate disorders | PRO170_mcid | TM |
| 29 | Psoriasis or eczema | Psoriasis | AH (half) |
| 30 | Psychoactive substance misuse | Substance_misuse | AH |
| 31 | RA connective tissue disorders | Rheumatoid_arthritis | AH (half) |
| 32 | Schizophrenia bipolar | Schizophrenia | AH |
| 32 | Schizophrenia bipolar | Bipolar_affective_disorder_and_mania | AH |
| 33 | Stroke transient ischaemic attack | Stroke_not_otherwise_specified | AH |
| 33 | Stroke transient ischaemic attack | Transient_ischaemic_attack | AH |
| 34 | Thyroid disorders | Tyroid_disease | AH |
| 35 | Myocardial Infarction | Myocardial_infarction | AH |
| **Demographic, lifestyle and test data** | | | |
| 36 | Ethnicity | Ethnicity_aurum_wgroups_mcid | Internet search |
| 37 | Body mass index | height | Self-generated |
| 37 | Body mass index | weight | Self-generated |
| 37 | Body mass index | bmi | Self-generated |
| 38 | Systolic blood pressure | sbp | Self-generated |
| 39 | Smoking status | cr_smokingcodes_aurum | Internet search |
| 40 | Cholesterol | chol_total | Self-generated |
| 40 | Cholesterol | chol_hdl | Self-generated |
| 40 | Cholesterol | chol_ratio | Self-generated |

Note that some of the code lists were edited after being moved onto incline as some formatting changes had to be made. I am keeping a table of the pre-formatted variable names for personal reference: (Table 2).

*Table SM3: Pre-formatted code list names and post-formatted code list names*

| **Variable** | **Pre-format name** | **Post-format name** |
| --- | --- | --- |
| Inflammatory bowel disease | IBD160_mapped | IBD160_mcid |
| Peptic Ulcer Disease | PEP135_mapped | PEP135_mcid |
| Prostate disorders | PRO170_mapped | PRO170_mcid |
| Ethnicity | Ethnicity_aurum_wgroups | Ethnicity_aurum_wgroups_mcid |
| Body mass index | height | Height_mcid |
| Body mass index | weight | Weight_mcid |
| Body mass index | bmi | bmi_mcid |
| Systolic blood pressure | sbp | sbp_mcid |
| Smoking status | cr_smokingcodes_aurum | cr_smokingcodes_aurum_mcid |
| Cholesterol | chol_total | chol_total_mcid |
| Cholesterol | chol_hdl | chol_hdl_mcid |
| Cholesterol | chol_ratio | chol_ratio_mcid |

## Code lists used for HES/ONS extraction

Table SM4 contains the variables and ICD 10 codes used to extract it. For chronic kidney disease, the full 5 digit ICD 10 code is required to separate CKD stages 1 and 2 from stages 3, 4 and 5. All others only require the initial 3 digits for extraction. Following the process in QRISK3, CHD and MI were grouped, and Stroke and TIA were grouped into one outcome.

*Table SM4: ICD 10 codes used for extraction*

| Variable | ICD 10 codes | # characters |
| --- | --- | --- |
| AF | 'I48' | 3 |
| CHD/MI | 'I20','I21','I22','I23','I24','I25' | 3 |
| Stroke/TIA | 'G45','I63','I64' | 3 |
| Heart failure | 'I50' | 3 |
| Type 1 diabetes | 'E10' | 3 |
| Type 2 diabetes | 'E11' | 3 |
| Chronic kidney disease stage 3/4/5 | 'N18.3','N18.30','N18.31','N18.32','N18.4','N18.5','N18.6','N18.9' | 5 |

# Multiple imputation methodology and evaluation of imputed data

## Methods

We used multiple imputation by chained equations to impute missing data for BMI, SBP, cholesterol/HDL ratio, smoking status, ethnicity and IMD. All variables listed in Table SM2 were included as predictors in the imputation procedure. Presence of variables in table SM4 at baseline were also included in the imputation procedure. The cohort was split into male and female cohorts and imputed separately (cohorts were then combined to fit analysis models). These imputation decisions would not be appropriate if developing this model for use in clinical practice. However, this is an illustrative example. The cohort used in this clinical example had been obtained as part of a larger grant, which will involve development of models which use all these other predictors, and will be developed separately for females and males. The imputation procedure was therefore carried out with this in mind, so the cohort could be used across multiple studies.

## Evaluation of imputed data

The level of missing data is given in Table SM5. Convergence was good for all imputed variables in both the male and female cohorts (see following two sections).

*Table SM5: Level of missing data*

|  | BMI | Chol/HDL ratio | Ethnicity | SBP | Smoking | IMD* |
| --- | --- | --- | --- | --- | --- | --- |
| male | 0.39 | 0.56 | 0.37 | 0.19 | 0.31 | 0 |
| female | 0.39 | 0.60 | 0.35 | 0.17 | 0.32 | 0 |

*the level of IMD missing data was 0% when rounded to 2 decimal places, but it did have missing data which was imputed in the imputation procedure

### Imputation of female cohort

## [1] "BMI convergence plot"


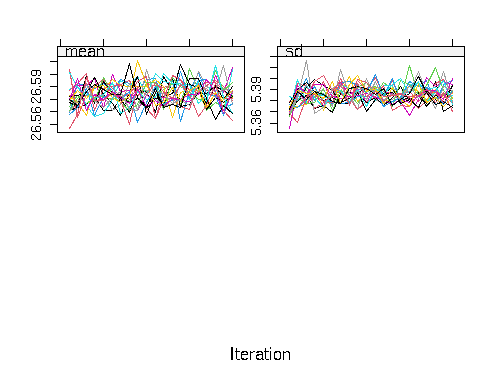


## [1] "BMI density plot"


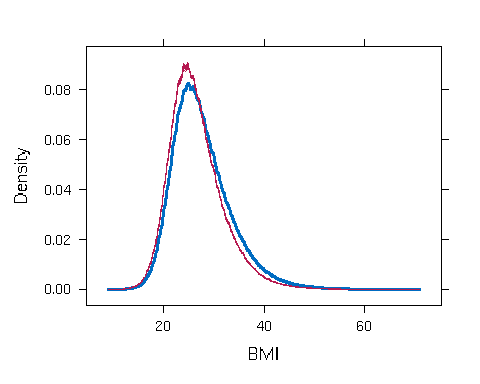


## [1] "Systolic blood pressure convergence plot"


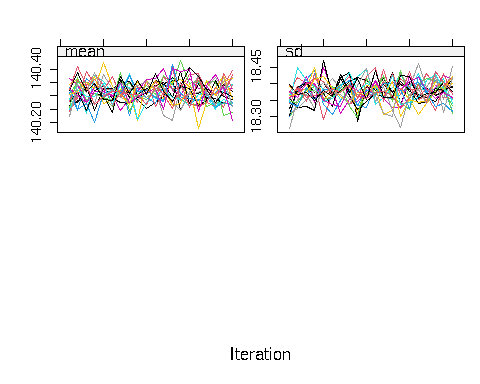


## [1] "Systolic blood pressure density plot"


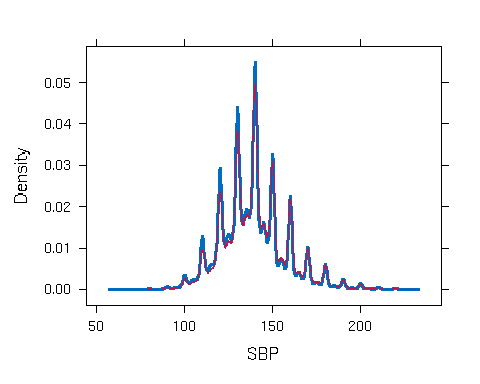


## [1] "Cholesterol convergence plot"


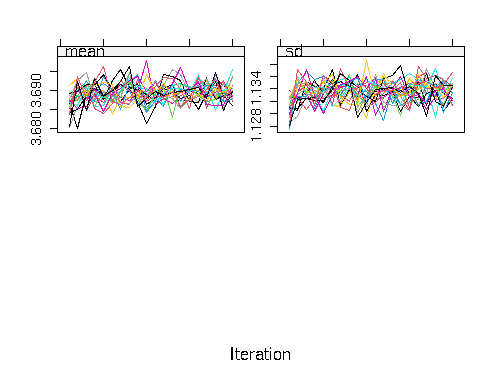


## [1] "Cholesterol density plot"


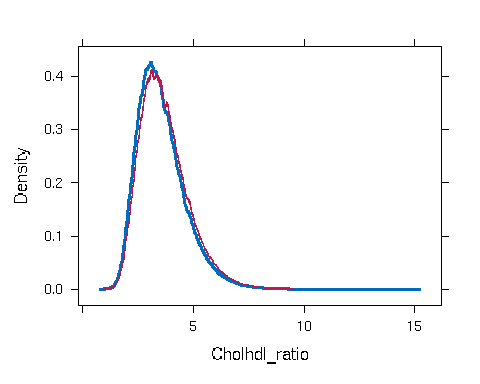


## [1] "Smoking Distribution"

## [1] "Non missing data"

| Var1 | Freq |
| --- | --- |
| Never | 41.19 |
| Ex | 31.56 |
| Current | 27.25 |

## [1] "Imputed data"

| Never | Ex | Current |
| --- | --- | --- |
| 46.74 | 26.85 | 26.42 |
| 46.72 | 26.93 | 26.36 |
| 46.65 | 26.97 | 26.38 |
| 46.72 | 26.93 | 26.35 |
| 46.76 | 26.93 | 26.31 |
| 46.76 | 26.92 | 26.32 |
| 47.01 | 26.80 | 26.20 |
| 46.75 | 26.92 | 26.33 |
| 46.82 | 26.80 | 26.37 |
| 46.77 | 26.88 | 26.36 |
| 46.81 | 26.83 | 26.36 |
| 46.71 | 26.87 | 26.42 |
| 46.83 | 26.81 | 26.37 |
| 46.89 | 26.85 | 26.27 |
| 46.85 | 26.83 | 26.32 |
| 46.79 | 26.89 | 26.32 |
| 46.91 | 26.72 | 26.37 |
| 46.79 | 26.93 | 26.28 |
| 46.89 | 26.90 | 26.21 |
| 46.83 | 26.92 | 26.25 |

## [1] "Ethnicity Distribution"

## [1] "Non missing data"

| Var1 | Freq |
| --- | --- |
| White | 92.86 |
| Mixed race | 0.56 |
| South asian | 3.47 |
| Black | 2.26 |
| Chinese and other | 0.85 |

## [1] "Imputed data"

| White | Mixed race | South asian | Black | Chinese and other |
| --- | --- | --- | --- | --- |
| 94.29 | 0.25 | 2.66 | 2.16 | 0.63 |
| 94.81 | 0.29 | 2.83 | 1.47 | 0.61 |
| 94.84 | 0.38 | 2.41 | 1.75 | 0.62 |
| 95.17 | 0.29 | 2.64 | 1.27 | 0.64 |
| 94.56 | 0.44 | 2.38 | 2.07 | 0.56 |
| 94.94 | 0.20 | 2.45 | 1.84 | 0.58 |
| 93.46 | 0.18 | 3.17 | 2.32 | 0.86 |
| 94.90 | 0.20 | 2.23 | 1.68 | 0.99 |
| 94.46 | 0.48 | 2.84 | 1.90 | 0.33 |
| 94.80 | 0.23 | 2.33 | 1.87 | 0.76 |
| 94.21 | 0.36 | 2.71 | 2.03 | 0.69 |
| 95.29 | 0.31 | 2.30 | 1.39 | 0.71 |
| 94.46 | 0.19 | 2.94 | 1.76 | 0.66 |
| 94.44 | 0.29 | 2.70 | 1.83 | 0.75 |
| 94.64 | 0.36 | 2.70 | 1.61 | 0.69 |
| 95.02 | 0.33 | 2.71 | 1.45 | 0.48 |
| 94.81 | 0.30 | 2.46 | 1.65 | 0.78 |
| 94.83 | 0.38 | 2.68 | 1.43 | 0.68 |
| 95.02 | 0.51 | 2.42 | 1.52 | 0.53 |
| 94.92 | 0.49 | 2.37 | 1.52 | 0.71 |

## [1] "IMD Distribution"

## [1] "Non missing data"

| Var1 | Freq |
| --- | --- |
| 1 (most deprived) | 24.52 |
| 2 | 22.42 |
| 3 | 20.19 |
| 4 | 17.79 |
| 5 (least deprived) | 15.08 |

## [1] "Imputed data"

| 1 (most deprived) | 2 | 3 | 4 | 5 (least deprived) |
| --- | --- | --- | --- | --- |
| 27.16 | 19.43 | 20.26 | 18.30 | 14.85 |
| 23.63 | 23.11 | 19.50 | 17.78 | 15.98 |
| 24.98 | 24.08 | 20.71 | 16.20 | 14.03 |
| 23.86 | 24.68 | 19.65 | 17.25 | 14.55 |
| 24.01 | 22.96 | 20.11 | 16.65 | 16.28 |
| 23.33 | 23.86 | 20.41 | 17.93 | 14.48 |
| 22.58 | 23.18 | 22.13 | 16.58 | 15.53 |
| 24.53 | 21.91 | 22.36 | 17.40 | 13.80 |
| 23.78 | 22.73 | 20.48 | 18.30 | 14.70 |
| 26.41 | 21.01 | 19.13 | 18.08 | 15.38 |
| 26.11 | 22.58 | 19.05 | 15.83 | 16.43 |
| 22.43 | 22.81 | 21.31 | 18.68 | 14.78 |
| 24.08 | 23.26 | 19.73 | 18.53 | 14.40 |
| 23.26 | 21.91 | 19.88 | 19.13 | 15.83 |
| 22.96 | 22.88 | 21.38 | 18.15 | 14.63 |
| 22.81 | 23.18 | 21.76 | 16.80 | 15.45 |
| 25.58 | 22.36 | 19.80 | 18.08 | 14.18 |
| 23.33 | 25.58 | 21.01 | 16.80 | 13.28 |
| 23.33 | 22.06 | 22.06 | 18.60 | 13.95 |
| 26.86 | 23.26 | 19.05 | 16.28 | 14.55 |

### Imputation of male cohort

## [1] "BMI convergence plot"


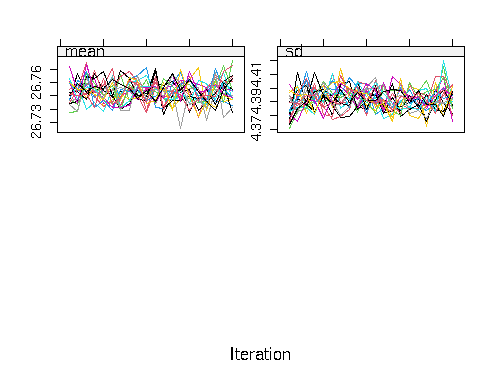


## [1] "BMI density plot"


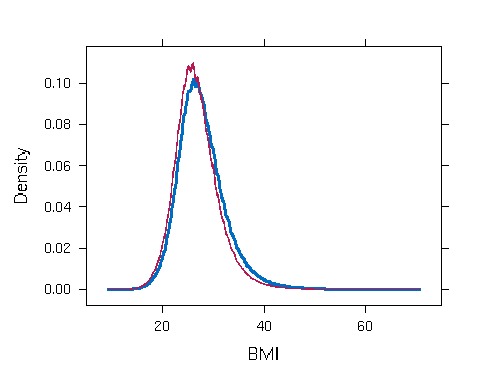


## [1] "Systolic blood pressure convergence plot"


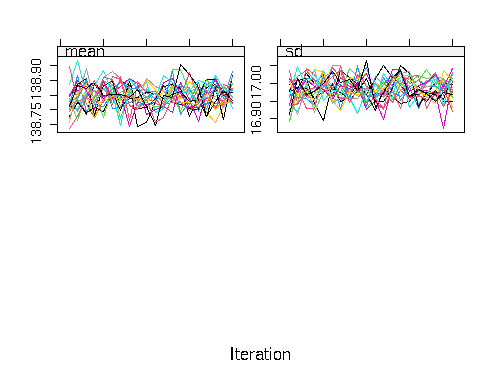


## [1] "Systolic blood pressure density plot"


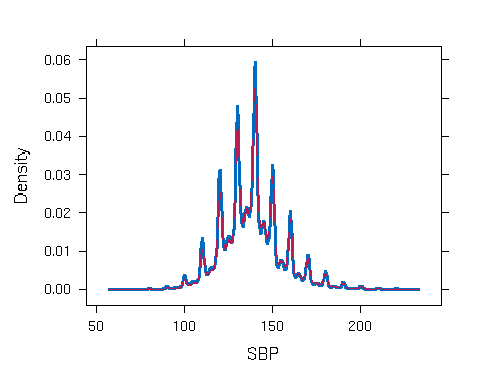


## [1] "Cholesterol convergence plot"


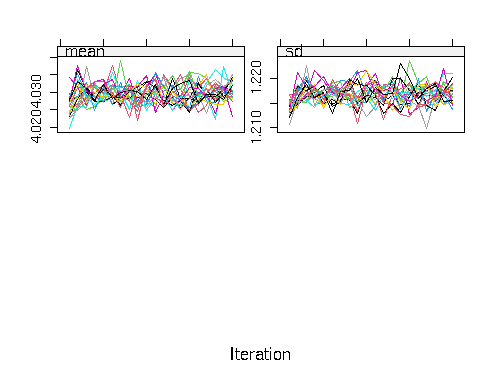


## [1] "Cholesterol density plot"


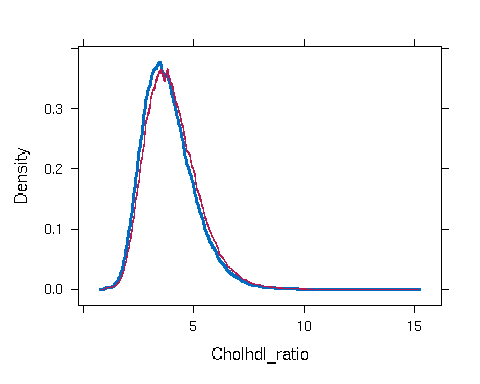


## [1] "Smoking Distribution"

## [1] "Non missing data"

| Var1 | Freq |
| --- | --- |
| Never | 27.16 |
| Ex | 43.95 |
| Current | 28.89 |

## [1] "Imputed data"

| Never | Ex | Current |
| --- | --- | --- |
| 30.84 | 39.27 | 29.89 |
| 30.93 | 39.26 | 29.81 |
| 30.87 | 39.32 | 29.81 |
| 30.94 | 39.13 | 29.92 |
| 30.87 | 39.37 | 29.76 |
| 30.90 | 39.23 | 29.87 |
| 30.93 | 39.23 | 29.85 |
| 30.99 | 39.24 | 29.76 |
| 30.82 | 39.26 | 29.92 |
| 30.76 | 39.42 | 29.83 |
| 30.94 | 39.31 | 29.75 |
| 30.80 | 39.36 | 29.84 |
| 30.94 | 39.17 | 29.89 |
| 30.95 | 39.21 | 29.84 |
| 30.98 | 39.28 | 29.73 |
| 30.99 | 39.36 | 29.66 |
| 30.83 | 39.24 | 29.93 |
| 30.99 | 39.16 | 29.85 |
| 30.67 | 39.37 | 29.96 |
| 30.96 | 39.28 | 29.77 |

## [1] "Ethnicity Distribution"

## [1] "Non missing data"

| Var1 | Freq |
| --- | --- |
| White | 92.96 |
| Mixed race | 0.51 |
| South asian | 3.64 |
| Black | 2.09 |
| Chinese and other | 0.80 |

## [1] "Imputed data"

| White | Mixed race | South asian | Black | Chinese and other |
| --- | --- | --- | --- | --- |
| 94.40 | 0.26 | 3.02 | 1.76 | 0.56 |
| 93.42 | 0.43 | 3.81 | 1.81 | 0.53 |
| 93.70 | 0.20 | 3.60 | 1.64 | 0.87 |
| 94.39 | 0.45 | 3.41 | 1.37 | 0.38 |
| 94.22 | 0.34 | 3.68 | 1.31 | 0.45 |
| 94.79 | 0.40 | 2.89 | 1.48 | 0.45 |
| 93.42 | 1.23 | 3.27 | 1.62 | 0.45 |
| 94.01 | 0.27 | 3.22 | 1.58 | 0.92 |
| 94.35 | 0.17 | 2.90 | 2.03 | 0.55 |
| 93.55 | 0.30 | 3.25 | 2.25 | 0.65 |
| 94.00 | 0.15 | 3.01 | 1.96 | 0.88 |
| 93.83 | 0.58 | 3.19 | 1.71 | 0.69 |
| 94.28 | 0.26 | 3.25 | 1.64 | 0.57 |
| 93.70 | 0.63 | 3.51 | 1.33 | 0.83 |
| 93.88 | 0.31 | 3.81 | 1.58 | 0.42 |
| 93.96 | 0.50 | 3.37 | 1.57 | 0.59 |
| 94.11 | 0.20 | 3.17 | 1.77 | 0.75 |
| 94.33 | 0.33 | 3.18 | 1.70 | 0.46 |
| 94.62 | 0.31 | 3.05 | 1.41 | 0.61 |
| 94.43 | 0.18 | 3.47 | 1.41 | 0.51 |

## [1] "IMD Distribution"

## [1] "Non missing data"

| Var1 | Freq |
| --- | --- |
| 1 (most deprived) | 24.72 |
| 2 | 22.59 |
| 3 | 20.19 |
| 4 | 17.40 |
| 5 (least deprived) | 15.10 |

## [1] "Imputed data"

| 1 (most deprived) | 2 | 3 | 4 | 5 (least deprived) |
| --- | --- | --- | --- | --- |
| 24.00 | 23.62 | 19.02 | 18.57 | 14.79 |
| 24.75 | 25.06 | 20.45 | 15.02 | 14.72 |
| 21.89 | 25.13 | 20.91 | 18.04 | 14.04 |
| 26.04 | 20.98 | 20.00 | 18.11 | 14.87 |
| 22.79 | 23.55 | 19.47 | 18.57 | 15.62 |
| 24.15 | 24.38 | 21.21 | 16.00 | 14.26 |
| 24.30 | 20.83 | 21.06 | 18.79 | 15.02 |
| 23.32 | 22.04 | 21.74 | 18.19 | 14.72 |
| 23.70 | 22.94 | 22.19 | 15.77 | 15.40 |
| 24.98 | 21.21 | 22.11 | 16.53 | 15.17 |
| 22.79 | 24.45 | 20.08 | 17.28 | 15.40 |
| 23.47 | 22.42 | 21.06 | 17.28 | 15.77 |
| 23.77 | 24.23 | 19.77 | 17.74 | 14.49 |
| 23.25 | 20.68 | 24.30 | 16.00 | 15.77 |
| 25.43 | 22.04 | 21.96 | 19.09 | 11.47 |
| 24.45 | 22.72 | 19.70 | 17.51 | 15.62 |
| 24.75 | 25.13 | 18.57 | 18.64 | 12.91 |
| 24.30 | 23.85 | 20.00 | 16.45 | 15.40 |
| 26.04 | 21.74 | 19.92 | 19.25 | 13.06 |
| 27.02 | 21.89 | 18.87 | 16.75 | 15.47 |

# Details of normal and gamma frailty models, priors, initial values and convergence plots

Full code for fitting the frailty models is available on GitHub REF XXXX.^1^ The definition of the models was common in both the simulation and the clinical example. The initial values and convergence plots provided here correspond to the clinical example.

## Normal frailty model

### Definition of model and priors

The following definition is in rtsan code:

model {

betas_A ~ normal(0,2);

betas_B ~ normal(0,2);

intercept_A ~ normal(-3,2);

intercept_B ~ normal(-3,2);

omega ~ normal(0,frail_param);

frail_param ~ cauchy(0, 2.5);

target += weibull_lpdf(times_uncens_A | shape_A, exp(-omega[IDs_uncens_A] - intercept_A - (X_uncens_A*betas_A)));

target += weibull_lccdf(times_cens_A | shape_A, exp(-omega[IDs_cens_A] - intercept_A - (X_cens_A*betas_A)));

target += weibull_lpdf(times_uncens_B | shape_B, exp(-omega[IDs_uncens_B] - intercept_B - (X_uncens_B*betas_B)));

target += weibull_lccdf(times_cens_B | shape_B, exp(-omega[IDs_cens_B] - intercept_B - (X_cens_B*betas_B)));

}

### Initial values

Initial values for each MCMC were sampled from the following distributions:

set_inits_normal <- function(seed = 1){

set.seed(seed)

return(list(shape_A = runif(1, 0.5, 1.5),

intercept_A = runif(1, -16, -13),

betas_A = c(runif(1, -0.1, 0.1), runif((stan_data$P - 1), -1.5, 1.5)),

shape_B = runif(1, 0.5, 1.5),

intercept_B = runif(1, -13, -9),

betas_B = c(runif(1, -0.1, 0.1), runif((stan_data$P - 1), -1.5, 1.5)),

frail_param = runif(1, 0.5, 1.5))

)

}

### Convergence plots

*Figure SM1: Convergence plot for predictor effects in normal frailty model*


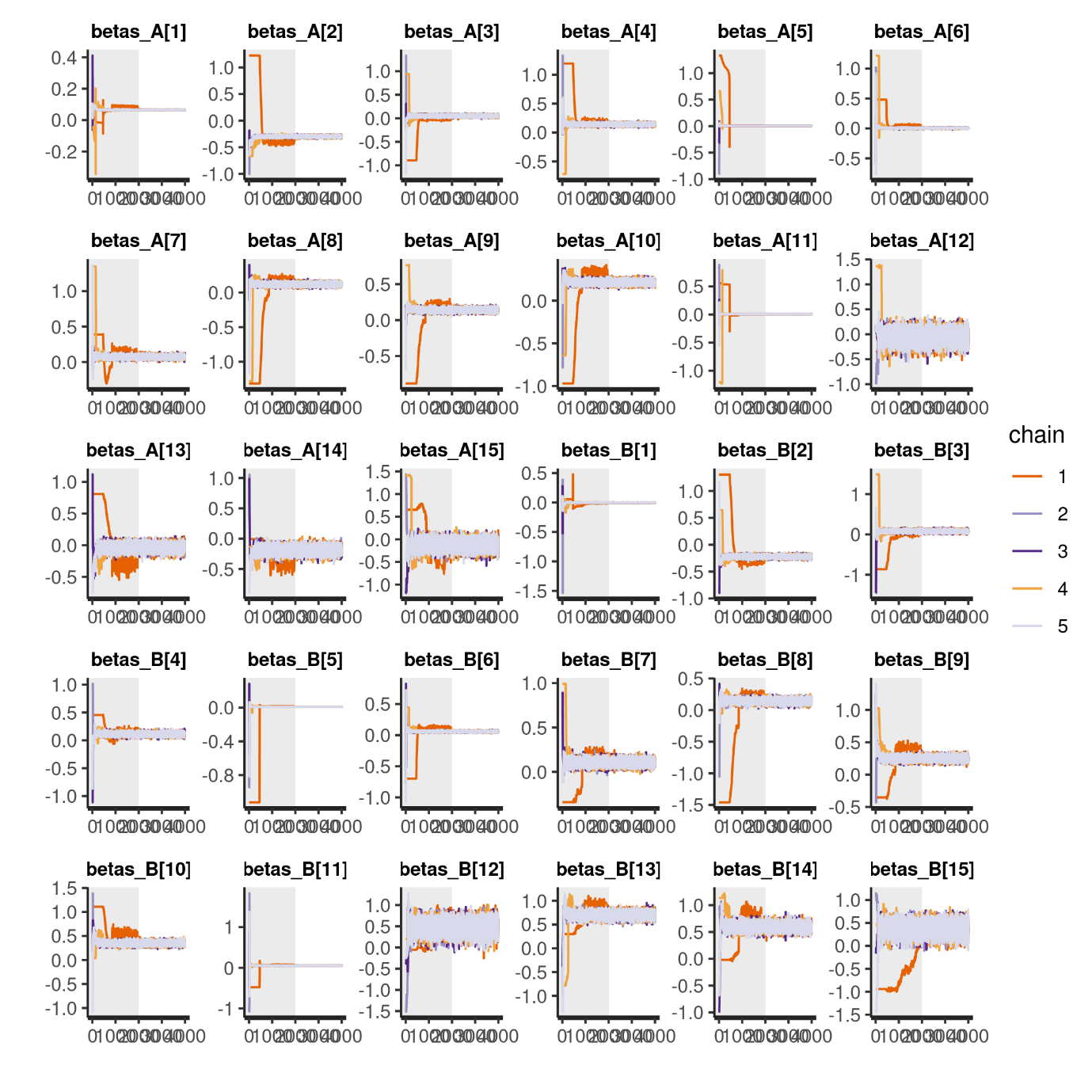


*Figure SM2: Convergence plots for shapes, scales and frailty parameter in normal frailty model*


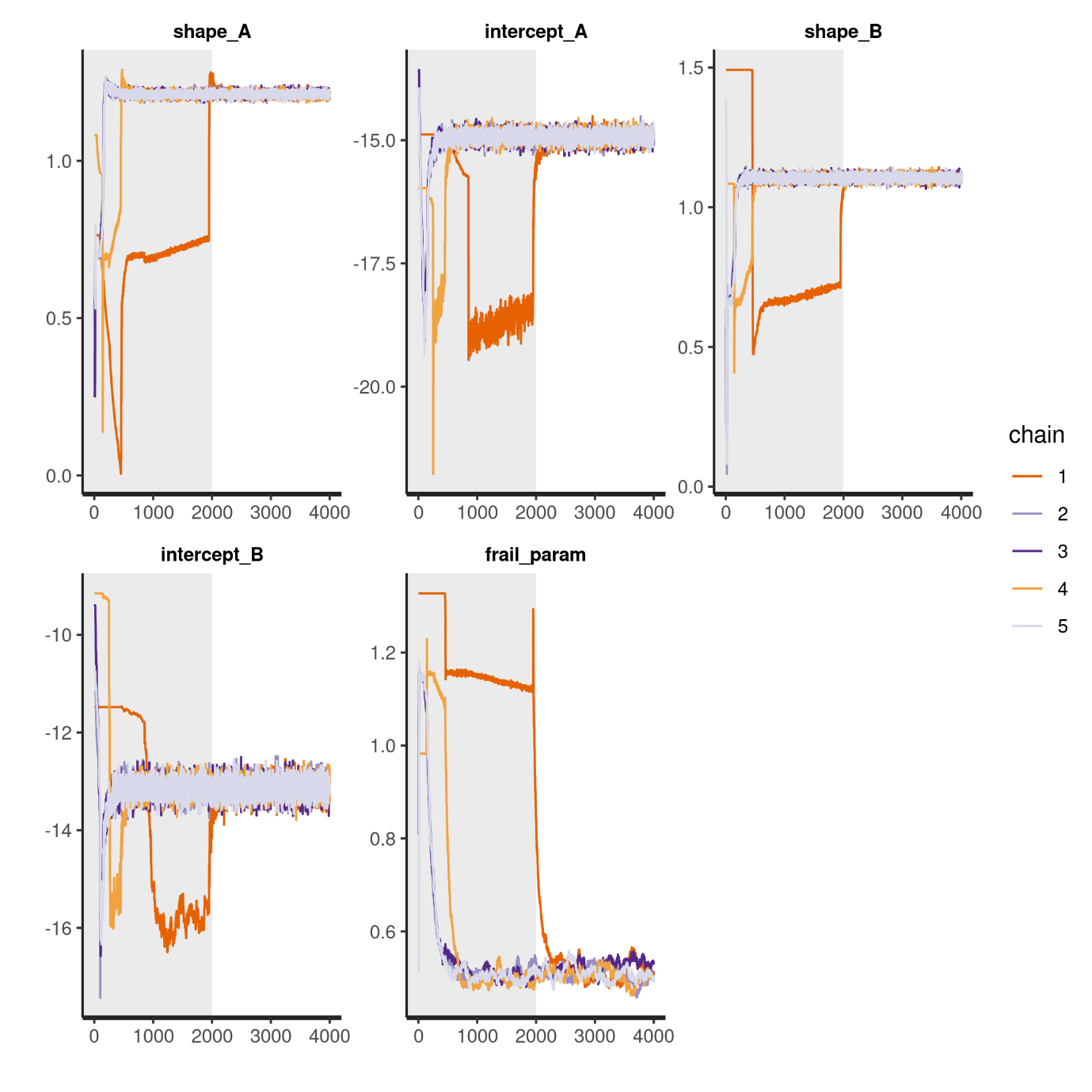


## Gamma frailty model

### Definition of model and priors

model {

betas_A ~ normal(0,1);

betas_B ~ normal(0,1);

intercept_A ~ normal(-7.5,2);

intercept_B ~ normal(-7.5,2);

omega ~ gamma(frail_param, frail_param);

frail_param ~ gamma(1,1);

target += weibull_lpdf(times_uncens_A | shape_A, exp(-log(omega[IDs_uncens_A]) - intercept_A - (X_uncens_A*betas_A)));

target += weibull_lccdf(times_cens_A | shape_A, exp(-log(omega[IDs_cens_A]) - intercept_A - (X_cens_A*betas_A)));

target += weibull_lpdf(times_uncens_B | shape_B, exp(-log(omega[IDs_uncens_B]) - intercept_B - (X_uncens_B*betas_B)));

target += weibull_lccdf(times_cens_B | shape_B, exp(-log(omega[IDs_cens_B]) - intercept_B - (X_cens_B*betas_B)));

}

### Initial values

Initial values for each MCMC were sampled from the following distributions:

set_inits_gamma <- function(seed = 1){

set.seed(seed)

return(list(shape_A = runif(1, 0.5, 1.5),

intercept_A = runif(1, -16, -13),

betas_A = c(runif(1, -0.1, 0.1), runif((stan_data$P - 1), -1.5, 1.5)),

shape_B = runif(1, 0.5, 1.5),

intercept_B = runif(1, -13, -9),

betas_B = c(runif(1, -0.1, 0.1), runif((stan_data$P - 1), -1.5, 1.5)),

frail_param = runif(1, 0.5, 2))

)

}

### Convergence plots

*Figure SM3: Convergence plot for predictor effects in gamma frailty model*


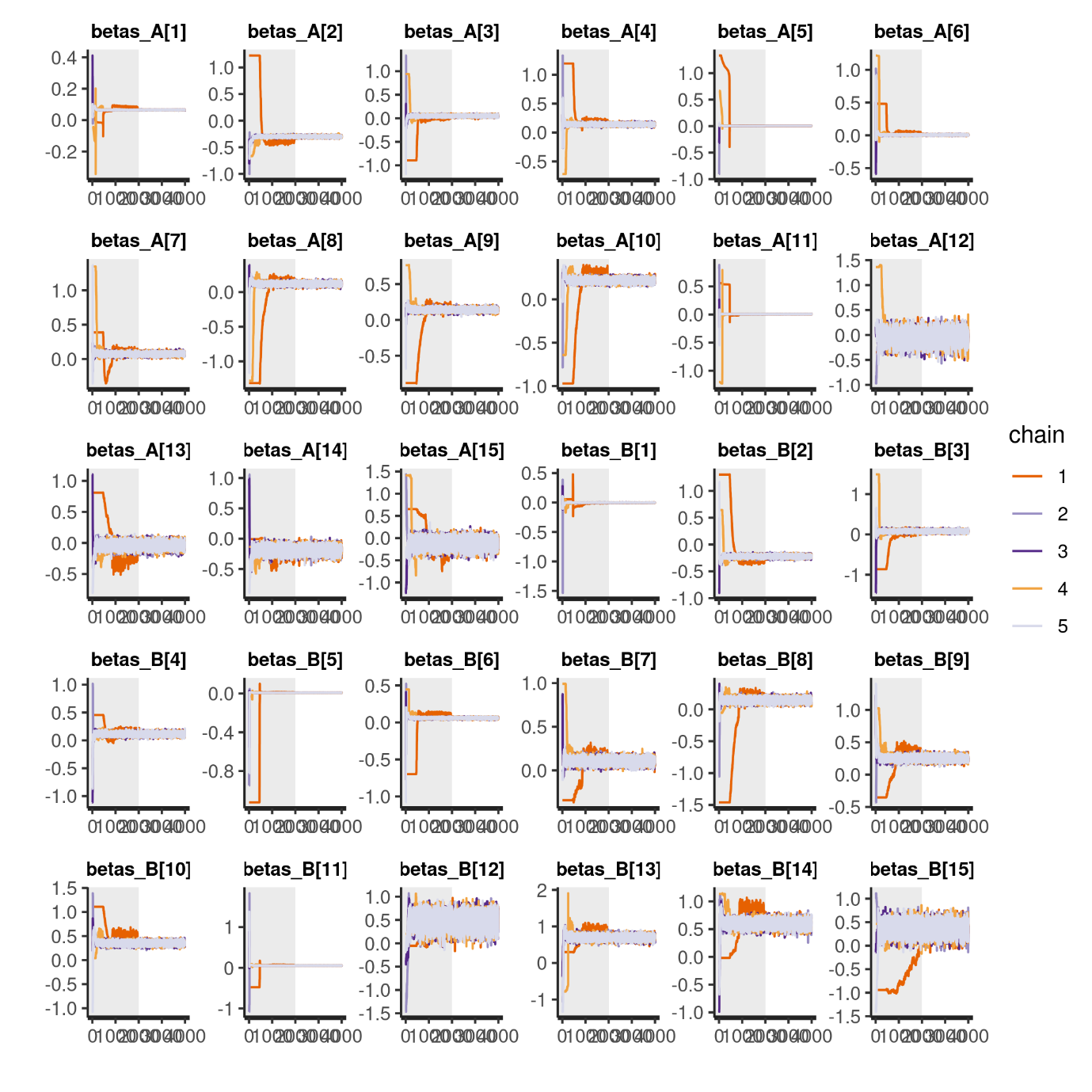


*Figure SM4: Convergence plots for shapes, scales and frailty parameter in gamma frailty model*


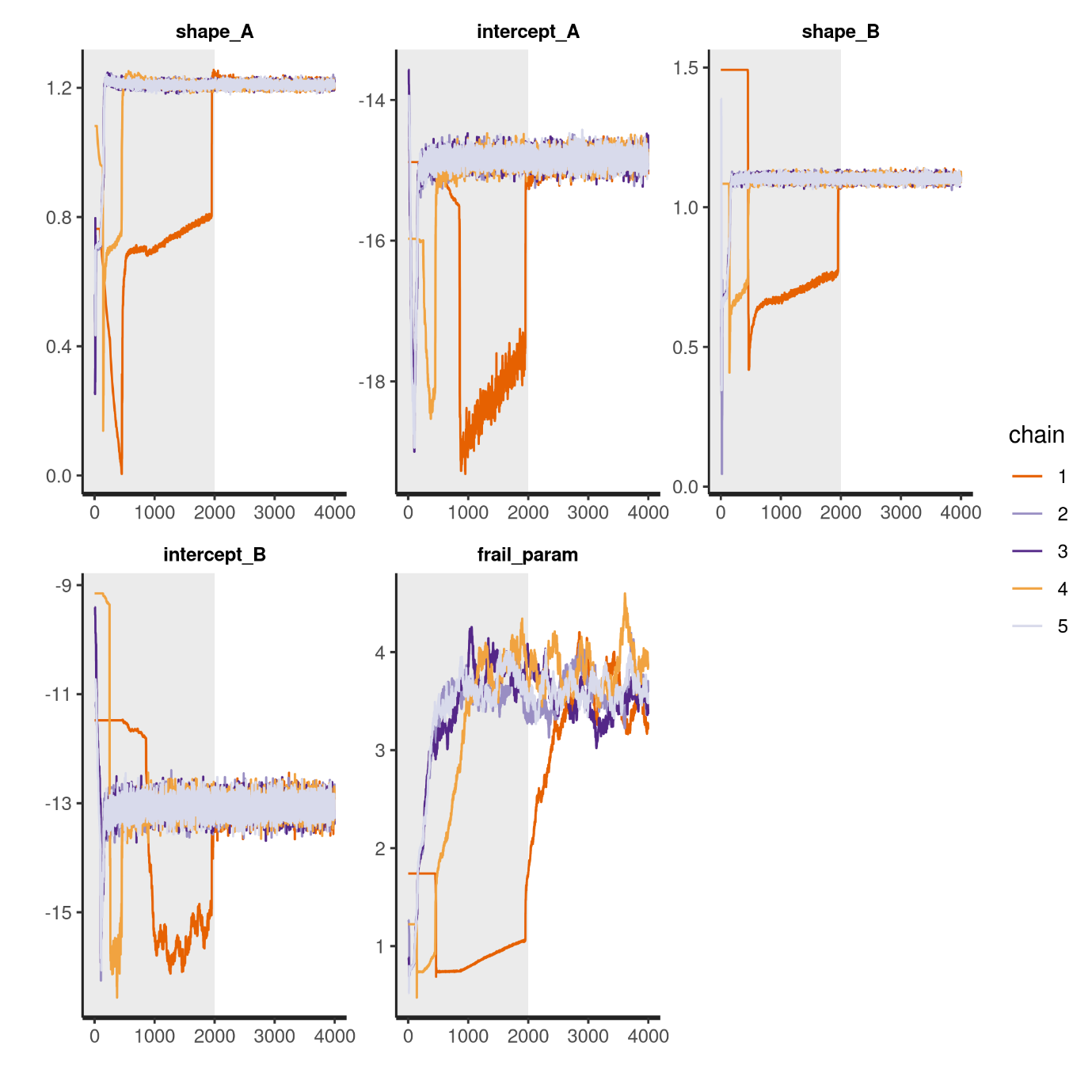

Supplement: Supplementary file 1 — Supporting information file 1. Supplementary material on methodology. [file SIM-42-3184-s002.docx]
